# The efficacy of a single dose of oral azithromycin in labour to prevent infections in infants and birthing parents in Fiji: secondary outcomes from a randomised controlled trial

**DOI:** 10.1136/bmjgh-2025-019851

**Published:** 2026-03-04

**Authors:** Maeve Hume-Nixon, Stephanie Clark, Tupou Ratu, Cattram Nguyen, Eleanor F G Neal, Kathryn Bright, Aneley Getahun Strobel, Emma Watts, John Hart, James J Fong, Eric Rafai, Kelera Sakumeni, Andrew Steer, Ilisapeci Vereti, Fiona Russell

**Affiliations:** 1 Department of Paediatrics, The University of Melbourne, Melbourne, Victoria, Australia; 2 Asia-Pacific Health, Murdoch Children’s Research Institute, Parkville, Victoria, Australia; 3 Ministry of Health and Medical Services, Suva, Fiji; 4 New Vaccines, Murdoch Children’s Research Institute, Parkville, Victoria, Australia; 5 Department of Microbiology and Immunology, The Peter Doherty Institute for Infection and Immunity, Melbourne, Victoria, Australia; 6 Tropical Diseases Research Group, Murdoch Children’s Research Institute, Parkville, Victoria, Australia; 7 Department of General Medicine, Royal Children's Hospital, Melbourne, Victoria, Australia

**Keywords:** Global Health, Maternal health, Paediatrics, Child health, Infections, diseases, disorders, injuries

## Abstract

**Introduction:**

Our Bulabula MaPei trial of azithromycin administered during labour in Fiji found no evidence of a reduction in the primary endpoint of infant skin and soft tissue infections (SSTIs) at 3 months of age. Here, we determine the efficacy of this intervention on several secondary outcomes.

**Methods:**

This randomised controlled trial included healthy pregnant adults presenting to hospital in labour. Prior to delivery, participants were randomly assigned a single dose of 2 g of oral azithromycin or placebo that were identical in appearance to mask treatment allocation, in a 1:1 ratio stratified by ethnicity. Cumulative incidence of infections and antibiotic prescription was compared using an intention-to-treat analysis of complete cases. Adverse events described as proportions by group at specified time points.

**Results:**

From June 2019 to January 2022, we enrolled 2110 pregnant people and their infants (n=2122; azithromycin n=1059; placebo n=1063). At 3 months, the cumulative incidence of infant infections was 13.6% in the azithromycin group compared with 17.3% in the placebo group (risk ratio (RR) 0.79; 95% CI 0.63 to 0.99; p=0.038). Infections among birthing parents, including SSTIs, were reduced with the greatest effect 1 week postdelivery (infections: RR 0.31; 95% CI 0.13 to 0.71; p=0.006, SSTIs: RR 0.25; 95% CI 0.08 to 0.75; p=0.013) but with a diminishing effect up to 6 months postdelivery. There was no effect on the prescription of antibiotics at any time point.

**Conclusions:**

Intrapartum azithromycin prevents a variety of infections for birthing parents and infants up to 12 months post partum in Fiji. However, further research is required to identify target populations and better characterise potential impacts on antimicrobial resistance and the infant microbiome and resistome.

**Trial registration number:**

NCT03925480.

**WHAT IS ALREADY KNOWN ON THIS TOPIC**

**WHAT THIS STUDY ADDS**

- This study, the first clinical trial of intrapartum oral azithromycin in the Western Pacific Region, extended follow-up beyond the neonatal and postpartum period, demonstrating that this intervention prevented infections in birthing parents, including skin and soft tissue infections, up to 6 months post partum, and infant infections up to 3 months.

**HOW THIS STUDY MIGHT AFFECT RESEARCH, PRACTICE OR POLICY**

- The relatively low number needed to treat to prevent infections in birthing parents suggests that use of prophylactic intrapartum azithromycin may be of benefit in some contexts; especially adjunctive prophylaxis in caesarean section, but there is limited evidence on potential impacts on antimicrobial resistance, and the infant microbiome and resistome.
- Given stagnated progress in improving maternal and child health and infection-related morbidity in some countries, its use may be considered as a temporary solution while broader, sustainable solutions are developed.

## Introduction

 Overall infections are the largest contributor to mortality in children younger than 5 years of age, responsible for approximately 43% of the estimated 5.1 million deaths per year and 18% of 2.4 million neonatal deaths each year.^1 2^ Furthermore, an estimated five million cases of pregnancy-related infections occur each year, resulting in 75 000 deaths.^3^ Aetiology of infant sepsis is age and setting dependent, but in low-income and middle-income countries (LMICs) common bacterial aetiology includes *Escherichia coli*, *Staphylococcus aureus*, *Streptococcus pneumoniae*, *Klebsiella pneumoniae*, *Neisseria meningitidis* and *Streptococcus pyogenes*,^4^ and many of these organisms are also associated with common postpartum infections including endometritis, urinary tract infections and bloodstream infections. and wound infections (ie, caesarean section incision sites, tears and episiotomies). Moreover, during the perinatal period, vertical transmission of infections, including group B streptococcus (GBS), can cause chorioamnionitis and endometritis in birthing parents and lead to neonatal sepsis,^5^ as well as horizontal transmission through spread of potentially pathogenic bacteria carried in the nasopharynx of birthing parents through close contact such as breastfeeding.

Use of intrapartum intravenous benzylpenicillin in high-income countries has reduced GBS neonatal sepsis,^5 6^ although there is emerging evidence to suggest that intrapartum antibiotic use may also lead to an increase in infections in the first year of life due to disruptions in the young infant’s microbiome.^7^ Intrapartum azithromycin has emerged as a potential solution for preventing common infections in birthing parents and infants. Azithromycin is a broad-spectrum macrolide antibiotic with bacteriostatic activity against both Gram-positive and Gram-negative bacteria that commonly cause infections in birthing parents and infants, including *S. aureus* and *S. pyogenes* that are associated with skin and soft tissue infections (SSTIs).^8 9^ Both oral and parenteral forms of azithromycin have long half-lives and therefore can be given as once-daily dosing, with the drug present for 2–4 weeks post-treatment in decreasing concentrations over time.^10 11^ Therefore, use of oral azithromycin offers benefits for feasibility in different settings compared with use of intrapartum benzylpenicillin which requires more frequent dosing and intravenous administration. Additionally, azithromycin has been shown to have a prolonged half-life and high-sustained antibiotic levels in placental tissues, and is transferred in breast milk,^12^ and therefore, has the potential to prevent and treat perinatal infections.^13^

A 2015 proof-of-concept study from the Gambia demonstrating intrapartum azithromycin reduced carriage of GBS, *S. aureus* and *S. pneumoniae* with subsequent reductions in infections in birthing parents and infants, especially infant skin infections and mastitis.^14^ A 2016 USA study in which women undergoing non-elective caesarean section received standard antibiotic prophylaxis with adjunctive azithromycin found a decreased risk of maternal endometritis (3.8% vs 6.1%, p=0.02) and wound infection (2.4% vs 6.6%, p<0.001).^15^ Subsequent large multicountry randomised controlled trials (RCTs) including over 40 000 pregnant persons have found reductions in maternal sepsis and death, mastitis and puerperal fever, and newborn skin infections.^16 17^ A 2024 systematic review and meta-analyses synthesising these RCTs found that intrapartum azithromycin prevented maternal endometritis, surgical site infections and maternal sepsis, but showed little or no evidence for impact on maternal mortality or neonatal sepsis or death.^18^ Additionally, mass drug administration of azithromycin to infants aged 1–11 months in some sub-Saharan African settings reduced child mortality, with the highest mortality reductions in younger infants aged 1–5 months.^19 20^ However, there are ongoing concerns around the impact of prophylactic use of azithromycin on population-level antimicrobial resistance (AMR) in key human pathogens, the microbiome and the resistome.

In Fiji, there are higher rates of infant infections, such as meningitis, in comparison to other middle-income countries and high-income countries.^21 22^ Moreover, there are high rates of infections that can be passed vertically including *Chlamydia trachomatis* and *Neisseria gonorrhoeae*,^23^ as well as carriage of pathogenic bacteria associated with invasive disease such as *S. pneumonia*e.^24^ Lack of resources, including appropriate culture medium, means that universal GBS screening in pregnancy is not currently feasible in Fiji; however, intrapartum benzylpenicillin is recommended if GBS is identified in a late gestation screening culture, or if there is intrapartum fever, preterm onset of labour or prolonged rupture of membranes in a pregnant person with unknown GBS status.^25^ Fiji has a high burden of morbidity associated with SSTIs, as shown by recent global burden of disease data.^26^ Additionally, a 2021 study reported that the incidence of primary healthcare presentations with scabies, SSTIs or both in Fiji (108.3 presentations per 1000 person-years) was up to 10 times greater than observed in high-income countries, with especially high rates among children under 5.^27^ Fiji also has a high incidence of serious complications from SSTIs including hospitalisations and invasive disease, as well as a high case-fatality rate associated with these conditions.^28^

Given the high rates of skin and other infection rates and colonisation of potential pathogens in birthing parents and infants, international evidence suggested intrapartum azithromycin as a cheap, feasible intervention to improve maternal and child outcomes in this context. Therefore, we conducted an RCT in Fiji (Bulabula MaPei) on the efficacy of a single dose of azithromycin given to pregnant people in labour, with the primary outcome examining whether this intervention prevented SSTIs in infants up to 3 months of age.^29^ This study found no evidence that the intervention prevented SSTIs in the azithromycin group (risk ratio (RR): 0.73; 95% CI 0.51 to 1.06).^30^ This RCT was the first intrapartum azithromycin study to be conducted in the Western Pacific Region, and that followed up infection and adverse event (AE) outcomes to 1 year of age, allowing sample collection to examine longer-term microbiological outcomes on bacterial carriage, AMR rates and the microbiome, as well as associated clinical sequelae. We now report on the secondary outcomes of the RCT which included infant SSTIs beyond the primary endpoint of 3 months; other infant infections; infections in birthing parents (including SSTIs); incidence of antibiotic prescriptions for birthing parents and infants; and AEs up to 12 months after administration.

## Methods

### Study design

The protocol and primary results of this phase III blinded, randomised, placebo-controlled trial have been published previously.^29 30^ This study was conducted in Suva, Fiji, where the main ethnic groups are indigenous Fijians (iTaukei, 57%) and Fijians of Indian Descent (38%).^31^ Specifically, recruitment, randomisation and the initial visit occurred at the Colonial War Memorial Hospital, the largest tertiary hospital in Fiji where an estimated 46% of all births occur nationally.^32 33^ 2110 pregnant people were randomised to receive either a single 2 g oral dose of azithromycin or placebo in a 1:1 ratio, which was administered during labour, or immediately prior to delivery in the case of caesarean section. Follow-up visits occurred at maternal child health clinics in the wider Suva area. Postpartum participants and their infants were followed up in six visits over 12 months (online supplemental figure 1).

### Patient and public involvement

There was collaboration with Colonial War Memorial Hospital and maternal child health clinic staff who were not involved in the study, to support appropriate integration of study processes into these settings. Patients were not involved in the design, conduct, reporting or dissemination plans for this research. Results from this study will be disseminated back to study participants and collaborators in the Fijian Ministry of Health and Medical Services.

### Participants

Pregnant potential participants were initially approached by study midwives and nurses at an antenatal clinic, and if interested, a witnessed, written informed consent process was done. To be eligible, individuals were at least 18 years old, intending to deliver at the Colonial War Memorial Hospital, living in Greater Suva and expecting to be available for the 12-month duration of the study. Those with cardiac, renal or hepatic abnormalities, or taking specific drugs that may interact with azithromycin were excluded.

When consented individuals were admitted in labour, or immediately prior to delivery for those undergoing caesarean section, final eligibility, including willingness to participate, was reconfirmed prior to randomisation.

### Randomisation and masking

Initially, study staff were available for randomisation during standard office hours Monday to Friday; however, after 8 months this was expanded to 24 hours per day, 7 days a week, to increase the window where randomisation was possible.

A computer-generated randomisation list with permuted blocks of variable length, stratified by ethnicity (Indigenous Fijian vs other) was generated by an independent statistician. The investigational product was packaged in blister packs marked with randomisation numbers, which were kept separately, stratified by ethnicity. After confirming eligibility at admission for delivery, study staff allocated the next available randomisation number from the randomisation list based on the participant’s self-reported ethnicity. The matching blister pack was given to the participant. Despite study staff potentially being involved in treatment allocation as well as assessing primary outcome, all study staff and participants were blinded to treatment allocation, as the placebo and azithromycin tablets were identical. Prior to the database lock and study unblinding, the Statistical Analysis Plan was written.

### Procedures

Randomisation occurred during labour (or prior to delivery in the case of caesarean section), and participants were administered the blister pack containing the study drug; receiving either 2 g of oral azithromycin (four 500 mg tablets) or placebo. Laboratorios Cinfa (Spain) manufactured azithromycin. Idifarma (Spain) manufactured placebo tablets and repackaged both azithromycin and placebo into blister packs.

Following randomisation, there were six visits performed over 12 months. The first visit was performed in hospital, and subsequent visits occurred at maternal child health clinics (online supplemental figure 1). There were periods where the COVID-19 pandemic restricted face-to-face study visits, so in-person physical examination was not performed; instead, follow-up study visits were conducted via phone.

At initial visits, demographic and pregnancy information was collected. At each study visit, the birthing parent and infant had their axillary temperature measured and underwent examination for SSTIs. Participants were also asked about outpatient diagnoses of SSTIs and other conditions since the last visit, self-reported antibiotic use, AEs and recent hospitalisations. Infants were assessed for weight, length and clinical Integrated Management of Childhood Illness danger signs.^34^ Caregivers were asked screening questions for conditions associated with azithromycin, including hearing impairment throughout the duration of the study.^35^

### Outcomes

The secondary objectives reported in this analysis included:

- cumulative incidence of total infant infections (meningitis, sepsis, lower respiratory tract infection, SSTI, fever, diarrhoea, urinary tract infection and ophthalmia neonatorum);
- cumulative incidence of total maternal infections (mastitis, sepsis, postoperative wound infections, SSTI, fever, meningitis, pneumonia, abdominal or pelvic abscess, endometritis, urinary tract infection, pyelonephritis and chorioamnionitis);
- cumulative incidence of antibiotics prescribed in infants and birthing parents up to 12 months of age/postdelivery; and
- AEs for infants and birthing parents from 6 weeks of age/postdelivery.

Total SSTIs in infants included impetigo, furuncle, omphalitis, abscess, cellulitis and staphylococcal scalded skin syndrome, and in birthing parents were defined as impetigo, furuncle, abscess, cellulitis, mastitis and postoperative wound infection. These outcomes were based on participant self-report, hospitalisation data from electronic hospital records and/or physical assessment at study visits. See online supplemental text boxes 1,2 for definitions of individual infection outcomes.

AEs were elicited at each study visit (online supplemental figure 1) through participant self-report of symptoms and hospitalisation, and verified through search of electronic hospital records that allowed identification of other serious AEs (SAEs). See online supplemental text box 3 for further details and full definitions of AEs and SAEs.

### Statistical analysis

This study was powered for the primary outcome and sample size calculations have been described previously.^29^ Sample size calculations were not performed for the composite secondary outcomes reported here, nor for the individual outcomes that made these up.

Case report forms were entered into Research Electronic Data Capture hosted by Murdoch Children’s Research Institute and following cleaning,^36 37^ all statistical analyses were performed using Stata V.18.0.^38^ Participant characteristics were summarised by treatment allocation. Continuous variables were summarised using means and standard deviations (or medians and IQRs for non-symmetrical data), and categorical variables reported as frequencies and percentages.

All secondary outcomes were calculated using an intention-to-treat principle; analysing participants in groups as randomised, and using complete case analysis that included participants with observed SSTI data at all visits up to the specified time point. For withdrawn participants, data up to their date of withdrawal (and beyond up to twelve months of follow-up, if permission to continue to check hospital records up to had been provided on withdrawal) was included in this analysis. The outcomes were binary, with the proportion of those with a secondary outcome calculated for azithromycin and placebo groups at each time point (birth, day 7, 6 weeks, 3 months, 6 months, 12 months). For total infections, SSTIs and antibiotic prescription for infants and birthing parents, RRs and risk differences with 95% CIs and p values were calculated, estimated using binomial regression (using log and identity links, respectively) with the stratification variable (ethnicity) as a covariate. Descriptive statistics were provided for individual infections. AEs were similarly binary and defined as an occurrence for each event and were described as proportions in each arm at specified time points. The denominator used for AEs was all participants that received the allocated dose, irrespective of incomplete data, and included those that withdrew following administration. This denominator represented the total number of births (including stillbirths) for infant participants. Primary analyses were based on an intention-to-treat population.

Due to the impacts of the COVID-19 pandemic on medical records and study procedures, there were a variety of missing data affecting baseline characteristics and primary and secondary outcomes, so sensitivity analyses were performed to explore the effect of including cases with partially observed outcome data. We performed additional analyses to explore how variation in modes of assessment of SSTIs related to COVID-19 restrictions impacted diagnosis of SSTIs distributed across the two arms.

Safety data were reviewed regularly by an independent Data Safety Monitoring Committee, and the study was registered with Clinical Trials.gov (Preventing Young Infant Infections Using Azithromycin in Labour (PreYIAL) Trial—NCT03925480).

### Reflexivity

Early-career and senior researchers from Fiji and Australia were involved in study design and implementation (Online supplemental file 1).

## Results

**Figure 1:**
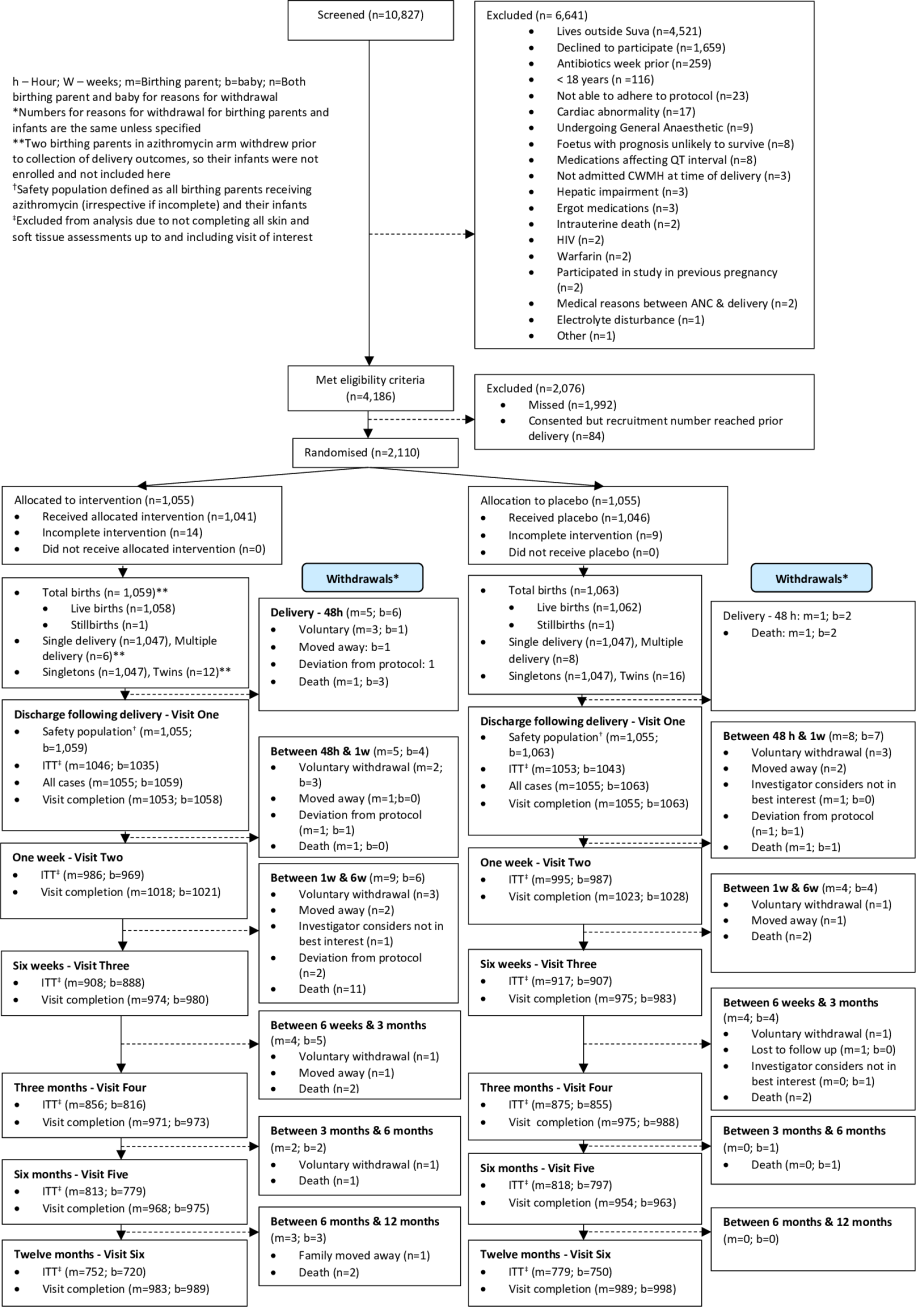
Participant flow diagram. ANC, Antenatal clinic; CWMH, Colonial War Memorial Hospital; ITT, intention-to-treat.

Recruitment occurred between 1 July 2019 and 31 January 2022. 10 827 pregnant persons were assessed for eligibility (figure 1). Following initial screening, 4186 remained eligible, and a total of 2110 were enrolled and randomised (1055 in each arm) with 1059 births in the azithromycin group and 1063 births in the placebo group. Recruitment was paused because of the COVID-19 pandemic for 10.5 months in total between 19 March 2020 and 6 November 2021. The final participant completed their last follow-up visit on 28 February 2023, marking trial completion. In total, 45 (2.1%) birthing parents and 44 (2.1%) infants were withdrawn, with more withdrawals in the azithromycin group compared with the placebo group (birthing parents: 28 vs 17; infants 26 vs 18, respectively). However, lower proportions of birthing parents and infants in the azithromycin arm reported that an AE factored into the decision to withdraw (birthing parents: 35.7% (azithromycin) vs 52.9% (placebo); infants: 34.6% (azithromycin) vs 55.6% (placebo). For infant participants, follow-up completion for individual visits was high, ranging from 91.4% to 100%. Despite this, the rate of complete case analysis; the proportion of participants that had skin assessments at all visits up to and including each specified time point, was comparatively low at later time points (lowest 69.3% at 12 months), with similar completion rates observed for birthing parents (see online supplemental tables 1,2).

Demographic and clinical characteristics were evenly distributed across groups, particularly risk factors for infection including exclusive breastfeeding, infant wasting and immunisation (table 1, online supplemental tables 3,4). Few infants were low birth weight (2.3%) or premature (3.0%). Birthing parents were mostly of iTaukei ethnicity (82.6%), 23.3% delivered via Caesarean section and 35.7% were treated with non-study antibiotics during admission.

**Table 1:** Characteristics of birthing parents and infants, by treatment allocation

	Azithromycin, n (%)*	Placebo, n (%)*
All birthing parents	N=1055	N=1055
Age in years, median (IQR)	27.4 (23.1–31.9)	27.4 (23.2–32.3)
Ethnicity		
Other	183 (17.3%)	184 (17.4%)
iTaukei/Indigenous Fijian	872 (82.7%)	871 (82.6%)
Residential location		
Rural	67 (6.4%)	67 (6.4%)
Urban	452 (42.8%)	436 (41.3%)
Periurban	536 (50.8%)	552 (52.3%)
Total number of household members, median (IQR) (N=2110)	6.0 (4.0–8.0)	6.0 (4.0–8.0)
Estimated weekly family income, $FJ median (IQR) (N=2107)	300.0 (200.0–400.0)	300.0 (200.0–400.0)
Cigarette use	103 (9.8%)	121 (11.5%)
Mode of delivery (N=2107)		
Vaginal—non-instrumental	807/1052 (76.7%)	791/1055 (75.0%)
Vaginal—instrumental	9/1052 (0.9%)	10/1055 (0.9%)
Caesarean section	236/1052 (22.4%)	254/1055 (24.1%)
Treated with other antibiotics during admission	355/1049 (33.8%)	396/1054 (37.6%)
Hours between rupture of membrane and delivery, median (IQR)	1.5 (0.2–6.3)	1.4 (0.2–6.9)
Hours between treatment and delivery, median (IQR) (N=2107)	10.2 (3.8–32.6)	11.2 (3.9–34.3)
72 hours or greater between administration of study drug and delivery	125/1052 (11.9%)	131/1055 (12.4%)
Multiple delivery (N=2107)†	6/1053 (0.6%)	8/1055 (0.8%)
All newborns	N=1059‡	N=1063
Sex (N=2121)		
Female	531/1058 (50.2%)	504/1063 (47.4%)
Apgar score at birth (N=2117)		
1–6	49/1056 (4.6%)	35/1061 (3.3%)
7–10	1007/1056 (95.4%)	1026/1061 (96.7%)
Birth weight (N=2106)		
Mean, grams (SD)	3441.0 (493.9)	3422.6 (496.8)
Low birth weight <2500 g	26/1050 (2.5%)	23/1056 (2.2%)
Gestational age (N=2109)		
Mean, weeks (SD)	39.7 (1.4)	39.5 (1.6)
Preterm birth (<37 w)	29/1051 (2.8%)	35/1058 (3.3%)
Exclusive breastfeeding		
At discharge following delivery	1043/1053 (99.1%)	1056/1062 (99.4%)
At 1 week	914/1011 (90.4%)	884/1019 (86.8%)
At 6 weeks	649/964 (67.3%)	652/970 (67.2%)
At 3 months	535/952 (56.2%)	540/972 (55.6%)
At 6 months	240/951 (25.2%)	233/946 (24.6%)
Wasting§		
At discharge following delivery	59/1013 (5.8%)	65/1022 (6.4%)
At 1 week	90/703 (12.8%)	95/732 (13.0%)
At 6 weeks	20/464 (4.3%)	24/480 (5.0%)
At 3 months	28/378 (7.4%)	22/428 (5.1%)
At 6 months	19/401 (4.7%)	24/409 (5.9%)
At 12 months	21/383 (5.5%)	12/385 (3.1%)
Received all vaccinations		
After birth	1040/1053 (98.8%)	1050/1062 (98.9%)
6-week vaccinations	963/968 (99.5%)	966/972 (99.4%)
10-week vaccinations	951/952 (99.9%)	964/972 (99.2%)
14-week vaccinations	941/952 (98.8%)	941/948 (99.3%)
12-month vaccinations	933/942 (99.0%)	945/957 (98.7%)

* Unless otherwise specified.

† Two birthing parents had missing delivery data as they were withdrawn prior to this being collected.

‡ One infant in the azithromycin group withdrew before baseline data were collected; therefore, 1058 was the denominator for most variables.

§ Using WHO child growth standards (based on age) wasted: weight-for-length z-scores <–2. Ref: Interpreting Growth Indicators. Geneva, Switzerland: WHO*; *2008.

### Infections in birthing parents

There was a lower cumulative incidence of infections overall and SSTIs at all time points in birthing parents who were administered azithromycin compared with placebo (table 2). At most time points, there was strong evidence of a reduction in infections, with the largest effect of a 69% reduction seen at 1 week postdelivery (RR 0.31; 95% CI 0.13 to 0.71; p=0.006), and a reduced effect at each subsequent time point with a 39% reduction seen at 6 months postdelivery (RR 0.61; 95% CI 0.42 to 0.88; p=0.008). This corresponded to an absolute risk reduction of between 1.8% and 3.6% and NNT for preventing one infection in birthing parents ranging from 28 to 56. A similar pattern was observed for SSTIs in birthing parents from 1 week postdelivery. There was strong evidence for a 75% reduction in SSTIs at 1 week postdelivery (RR 0.25; 95% CI 0.08 to 0.75; p=0.013), which reduced to 44% at 12 months postdelivery (RR 0.56; 95% CI 0.35 to 0.88; p=0.013) equating to an absolute reduction of between −1.3% and −2.9%, and NNT of 79 and 34.

**Table 2:** Cumulative incidence of all infections in birthing parents up to and including 12 months postdelivery by study arm

	Discharge following delivery					1 week postdelivery					6 weeks postdelivery				
	Azithromycin n/1046 (%)	Placebo n/1053 (%)	RR (95% CI)	P value	RD (%) (95% CI)	Azithromycin n/986 (%)	Placebo n/995 (%)	RR (95% CI)	P value	RD (%) (95% CI)	Azithromycin n/908 (%)	Placebo n/917 (%)	RR (95% CI)	P value	RD (%) (95% CI)
**Infections in birthing parents including SSTIs (total)**	3 (0.29)	5 (0.47)	0.60 (0.14 to 2.51)	0.49	−0.23 (−0.87 to 0.41)	7 (0.71)	23 (2.31)	0.31 (0.13 to 0.71)*	0.006*	−1.77 (−2.81 to −0.73)* **NNT 56 (36 to 137)**	19 (2.09)	42 (4.58)	0.46 (0.27 to 0.78)*	0.004*	−2.64 (−4.26 to −1.03)* **NNT 38 (23 to 97)**
Meningitis	0	0				0	1 (0.10)				0	1 (0.11)			
Sepsis	0	0				1 (0.10)	1 (0.10)				1 (0.11)	2 (0.22)			
Puerperal sepsis	0	0				0	0				0	0			
Pneumonia	0	1 (0.09)				0	1 (0.10)				0	0			
Abdominal or pelvic abscess	0	0				0	0				0	0			
Endometritis	0	0				0	0				0	0			
Urinary tract infection	0	0				0	1 (0.10)				0	1 (0.11)			
Pyelonephritis	0	0				0	0				0	0			
Chorioamnionitis	1 (0.10)	0				1 (0.10)	0				1 (0.11)	0			
Fever	2 (0.19)	2 (0.19)				3 (0.30)	7 (0.70)				9 (0.99)	14 (1.53)			
SSTIs in birthing parents (total)	1 (0.10)	3 (0.28)	0.33 (0.03 to 3.21)	0.343	−0.23 (−0.68 to 0.22)	4 (0.41)	16 (0.50)	0.25 (0.08 to 0.75)*	0.013*	−1.26 (−2.12 to −0.40)* **NNT 79 (47−250)**	10 (1.10)	30 (3.27)	0.34 (0.17 to 0.68)*	0.003*	−2.31 (−3.59 to −1.03)* **NNT 43 (28 to 97)**
Impetigo	0	1 (0.09)				0	0				0	0			
Furuncle	0	1 (0.09)				0	3 (0.30)				1 (0.11)	13 (1.42)			
Abscess	0	1 (0.09)				1 (0.10)	2 (0.20)				6 (0.66)	5 (0.55)			
Cellulitis	0	0				0	0				0	0			
Mastitis	0	0				0	1 (0.10)				0	2 (0.22)			
Postoperative wound infection	1 (0.10)	0				3 (0.30)	10 (1.01)				3 (0.33)	10 (1.31)			

	3 months postdelivery					6 months postdelivery					12 months postdelivery				
	Azithromycin n/856 (%)	Placebo n/875 (%)	RR (95% CI)	P value	RD (%) (95% CI)	Azithromycin n/813 (%)	Placebo n/818 (%)	RR (95% CI)	P value	RD (%) (95% CI)	Azithromycin n/752 (%BB)	Placebo n/779 (%)	RR (**95% CI**)	P value	RD (%) (**95% CI**)
**Infections in birthing parents including SSTIs (total)**	28 (3.27)	45 (5.14)	0.64 (0.40 to 1.01)	0.055	−2.05 (−3.91 to −0.18) **NNT 49 (26 to 556)**	43 (5.29)	71 (8.68)	0.61 (0.42 to 0.88)*	0.008*	−3.55 (−6.00 to −1.11)* **NNT 28 (17 to 90)**	105 (13.96)	138 (17.72)	0.79 (0.62 to 1.00)	0.045	−3.72 (−7.37 to −0.07)
Meningitis	0	1 (0.11)				0	1 (0.12)				0	1 (0.13)			
Sepsis	1 (0.12)	2 (0.23)				1 (0.12)	2 (0.24)				1 (0.13)	2 (0.26)			
Puerperal sepsis	0	0				0	0				0	0			
Pneumonia	2 (0.23)	1 (0.11)				3 (0.37)	4 (0.49)				5 (0.66)	8 (1.03)			
Abdominal or pelvic abscess	0	0				0	0				0	0			
Endometritis	0	0				0	0				0	0			
Urinary tract infection	1 (0.12)	1 (0.11)				2 (0.25)	1 (0.12)				2 (0.27)	2 (0.26)			
Pyelonephritis	0	0				0	0				0	0			
Chorioamnionitis	1 (0.12)	0				1 (0.12)	0				1 (0.13)	0			
Fever	13 (1.52)	18 (2.06)				26 (3.20)	39 (4.77)				83 (11.04)	101 (12.97)			
SSTIs in birthing parents (total)	17 (1.99)	36 (4.11)	0.48 (0.27 to 0.85)*	0.012*	−2.52 (−4.01 to −1.03)* **NNT 40 (25 to 99)**	22 (2.71)	44 (5.38)	0.50 (0.30 to 0.83)*	0.007*	−2.73 (−4.61 to −0.84)* **NNT 37 (22 to 119)**	27 (3.59)	50 (6.42)	0.56 (0.35 to 0.88)*	0.013*	−2.90 (−5.06 to −0.73)* **NNT 34 (20 to 137)**
Impetigo	0	0				0	2 (0.24)				0	2 (0.26)			
Furuncle	5 (0.58)	18 (2.06)				9 (1.11)	23 (2.81)				14 (1.86)	28 (3.59)			
Abscess	9 (1.05)	7 (0.80)				11 (1.35)	7 (0.86)				11 (1.46)	9 (1.16)			
Cellulitis	0	0				0	1 (0.12)				0	1 (0.13)			
Mastitis	0	2 (0.23)				0	2 (0.24)				0	2 (0.26)			
Postoperative wound infection															

Composite secondary outcome bolded. RRs and risk differences are reported for composite secondary outcomes. Individual infections are reported descriptively.

* 95% CI does not include null value. NNT presented for these results only.

NNT, number needed to treat; RD, risk difference; RR, risk ratio; SSTIs, skin and soft tissue infections.

### Infant infections

The proportion of infant infections in the azithromycin group was reduced at all time points compared with the placebo group except for at discharge following delivery (table 3). However, this difference was only statistically discernible at 3 months, with a 21% reduced risk of infant infections in the azithromycin arm compared with placebo (RR 0.79; 95% CI 0.63 to 0.99; p=0.038), corresponding to an absolute reduction of 3.7% and an NNT of 27 to prevent one infection. For infant SSTIs, there was little or no evidence of an effect at any time point (table 3).

**Table 3:** Cumulative incidence of all infant infections up to and including 12 months of age by study arm

	Discharge following delivery					1 week of age					6 weeks of age				
	Azithromycin n/1035* (%)	Placebo n/1043* (%)	RR (95% CI)	P value	RD (%) (95% CI)	Azithromycin n/969* (%)	Placebo n/987* (%)	RR (95% CI)	P value	RD (%) (95% CI)	Azithromycin n/888* (%)	Placebo n/907* (%)	RR (95% CI)	P value	RD (%) (95% CI)
**All infant infections including SSTIs (total**)	31 (3.00)	29 (2.78)	1.08 (0.65 to 1.77)	0.77	0.25 (−1.17 to 1.67)	43 (4.44)	46 (4.66)	0.95 (0.63 to 1.43)	0.81	−0.22 (−2.06 to 1.63)	67 (7.55)	78 (8.60)	0.88 (0.64 to 1.20)	0.41	−1.08 (−3.60 to 1.44)
Meningitis (total)	1 (0.10)	3 (0.29)				3 (0.31)	3 (0.30)				4 (0.45)	2 (0.45)			
Sepsis	3 (0.29)	1 (0.10)				6 (0.62)	4 (0.41)				4 (0.45)	3 (0.33)			
Lower respiratory tract infection	23 (2.22)	18 (2.22)				22 (2.27)	18 (1.82)				23 (2.59)	23 (2.54)			
Diarrhoea	0	0				1 (0.10)	4 (0.41)				4 (0.45)	6 (0.66)			
Urinary tract infection	3 (0.29)	5 (0.48)				5 (0.52)	4 (0.41)				8 (0.90)	7 (0.77)			
Ophthalmia neonatorum	0	0				0	0				0	1 (0.11)			
Fever	9 (0.87)	7 (0.67)				6 (0.62)	6 (0.61)				30 (3.38)	31 (3.42)			
Infant SSTIs (total)	1 (0.10)	2 (0.19)	0.50 (0.05 to 5.55)	0.58	−0.11 (−0.51 to 0.28)	4 (0.41)	12 (1.22)	0.34 (0.11 to 1.05)	0.061	−0.88 (−1.66 to −0.09)	15 (1.69)	24 (2.65)	0.64 (0.34 to 1.21)	0.17	−1.06 (−2.39 to 0.27)
Omphalitis	0	1 (0.10)				1 (0.10)	5 (0.51)				1 (0.11)	5/908 (0.55)			
Impetigo	0	0				0	2 (0.20)				3 (0.34)	4/907 (0.44)			
Furuncle	1 (0.10)	1 (0.10)				3 (0.31)	5 (0.51)				8 (0.90)	13 (1.43)			
Abscess	0	0				0	1 (0.10)				3 (0.34)	4 (0.44)			
Cellulitis	0	0				0	0				0	0			
SSSS	0	0				0	0				0	0			

	3 months of age					6 months of age					12 months of age				
	Azithromycin n/816* (%)	Placebo n/855* (%)	RR (95% CI)	P value	RD (%) (95% CI)	Azithromycin n/779* (%)	Placebo n/797* (%)	RR (95% CI)	P value	RD (%) (95% CI)	Azithromycin n/ 720* (%)	Placebo n/750* (%)	RR (95% CI)	P value	RD (%) (95% CI)
**All infant infections including SSTIs (total**)	111 (13.60)	148 (17.31)	0.79 (0.63 to 0.99)†	0.038†	−3.72 (−7.19 to −0.24)† **NNT 27 (14 to 417**)	223 (28.63)	265 (33.25)	0.86 (0.74 to 1.00)	0.044	−4.56 (−9.13 to 0.01)	387 (53.75)	435 (58.00)	0.92 (0.84 to 1.01)	0.088	−4.36 (−9.44 to 0.71)
Meningitis (total)	4 (0.49)	3 (0.35)				5 (0.64)	3 (0.38)				8 (1.11)	2 (0.27)			
Sepsis	4 (0.49)	3 (0.35)				4 (0.51)	4 (0.50)				5 (0.69)	5 (0.67)			
Lower respiratory tract infection	29 (3.55)	31 (3.63)				61 (7.83)	63 (7.90)				118 (16.39)	114 (15.20)			
Diarrhoea	4 (0.49)	10 (1.17)				14 (1.80)	17 (2.13)				27 (3.75)	31 (4.13)			
Urinary tract infection	8 (0.98)	7 (0.82)				9 (1.16)	5 (0.63)				7 (0.97)	5 (0.67)			
Ophthalmia neonatorum	0	1 (0.12)				0	1 (0.13)				0	1 (0.13)			
Fever	47 (5.76)	61 (7.13)				118 (15.15)	152 (19.07)				293 (40.69)	352 (46.93)			
Infant SSTIs (total)	46 (5.64)	67 (7.84)	0.72 (0.50 to 1.04)	0.079	−2.20 (−4.62 to 0.22)	95 (12.20)	95 (15.06)	0.81 (0.63 to 1.04)	0.093	−2.57 (−5.92 to 0.77)	142 (19.72)	160 (21.33)	0.92 (0.75 to 1.12)	0.39	−1.26 (−5.32 to 2.80)
Omphalitis	1 (0.12)	8/856 (0.93)													
Impetigo	10 (1.23)	14 (1.64)				30 (3.85)	30 (3.76)				43 (5.97)	45 (6.00)			
Furuncle	22 (0.03)	32 (3.74)				50 (6.42)	58 (7.28)				78 (10.83)	78 (10.40)			
Abscess	20/817 (2.45)	21 (2.46)				34 (4.36)	41 (5.14)				42/722 (5.82)	56/751 (7.46)			
Cellulitis	2 (0.25)	0				3 (0.39)	0				7/721 (0.97)	3/751 (0.40)			
SSSS	0	0				0	0				0	0			

Composite secondary outcome bolded. RRs and risk differences are reported for composite secondary outcomes. Individual infections are reported descriptively.

* Unless otherwise specified.

† 95% CI does not include null value. NNT presented for these results only.

NNT, number needed to treat; RD, risk difference; RR, risk ratio; SSSS, Staphylococcal scaled skin syndrome; SSTIs, skin and soft tissue infections.

### Antibiotic prescription in infants and birthing parents

There was a lack of evidence of any difference between groups in prescription of antibiotics at any time point, for infants or birthing parents (table 4).

**Table 4:** Cumulative incidence of antibiotic prescription in birthing parents and infants (throughout study duration)

	Discharge following delivery				1 week post delivery				6 weeks postdelivery				3 months postdelivery				6 months postdelivery				12 months postdelivery			
Infant participant prescribed antibiotics	**AZI n/1035 (%**)	**PLA n/1043 (%**)	**RR** **(95% CI**)	**P value**	**AZI n/969 (%**)	**PLA n/987 (%**)	**RR (95% CI**)	**P value**	**AZI n/888 (%**)	**PLA n/907 (%**)	**RR (95% CI**)	**P value**	**AZI n/816 (%**)	**PLA n/855 (%**)	**RR (95% CI**)	**P value**	**AZI n/779 (%**)	**PLA n/797 (%**)	**RR (95% CI**)	**P value**	**AZI n/720 (%**)	**PLA n/750 (%**)	**RR (95% CI**)	**P value**
	153 (14.8)	160 (15.3)	0.96 (0.79 to 1.18)	0.73	164 (16.9)	190 (19.3)	0.88 (0.73 to 1.06)	0.19	168 (18.9)	187 (20.6)	0.92 (0.76 to 1.11)	0.37	183 (22.4)	217 (25.4)	0.88 (0.74 to 1.05)	0.16	271 (34.8)	302 (37.9)	0.92 (0.80 to 1.05)	0.20	320 (44.4)	368 (49.1)	0.90 (0.81 to 1.00)	0.061
Birthing parent prescribed antibiotics	**AZI n/1046 (%)**	**PLA n/1053 (%)**	**RR** **(95% CI)**	**P value**	**AZI n/986 (%)**	**PLA n/995 (%)**	**RR (95% CI)**	**P value**	**AZI n/908 (%)**	**PLA n/917 (%)**	**RR (95% CI)**	**P value**	**AZI n/856 (%)**	**PLA n/875 (%)**	**RR (95% CI)**	**P value**	**AZI n/813 (%)**	**PLA n/818 (%)**	**RR (95% CI)**	**P value**	**AZI n/752 (%)**	**PLA n/779 (%)**	**RR (95% CI)**	**P value**
	355 (33.9)	396 (37.6)	0.90 (0.80 to 1.01)	0.079	348 (35.3)	389 (39.1)	0.90 (0.80 to 1.01)	0.078	333 (36.7)	369 (40.2)	0.91 (0.81 to 1.02)	0.11	317 (37.0)	364 (41.6)	0.89 (0.79 to 1.00)	0.05	314 (38.6)	350 (42.8)	0.90 (0.80 to 1.02)	0.087	298 (39.6)	349 (44.8)	0.89 (0.79 to 1.00)	0.044

AZI, azithromycin; PLA, placebo; RR, risk ratio.

### Sensitivity analyses

There were differences in characteristics of birthing parents that were included in complete case analysis, compared with those that were not included (ie, had incomplete observational data). Specifically, those not included were more likely to be of iTaukei ethnicity, use cigarettes and have a lower household income, and were less likely to have had a caesarean section (online supplemental table 5). After delivery prior to discharge, there were large differences in prematurity, low birth weight and low Apgar score between infants included in complete case analysis and those not included. However, these differences had mostly resolved by 3 months (online supplemental table 5). Sensitivity analyses were performed including individuals with partially observed outcome data, that is, the derived outcome variable included all cases rather than requiring complete SSTI assessment up to and including the specified time point. When this sensitivity analysis was performed for infants, it showed no evidence of an impact of azithromycin at 3 months (RR 0.86; 95% CI 0.71 to 1.05 p=0.134), and little evidence for an effect at 12 months of age (RR 0.92, 95% CI 0.85 to 0.99, p=0.034) (online supplemental table 6), contrasting with findings from complete case analysis. This sensitivity analysis showed little effect on the observed impact of intrapartum azithromycin on infections overall and SSTIs for birthing parents (online supplemental table 7). When all cases were included in the analysis of antibiotic prescription for infants, there was some evidence of a 10% reduction in prescription at 12 months in the azithromycin group (RR 0.90; 95% CI 0.82 to 0.99; p=0.04). For antibiotic prescription for birthing parents, this sensitivity analysis showed a reduction of approximately 11% at all visits from 1 week postdelivery, which contrasted with the lack of effect demonstrated in the complete case analysis (online supplemental table 8).

### AEs for infants and birthing parents

AEs up to 6 weeks, and SAEs up to 3 months of age/postdelivery have been described previously, showing azithromycin was generally well tolerated.^30^ Overall, SAEs in infants and birthing parents up to 12 months of follow-up were similar in both groups, and the majority were unrelated to the study drug (online supplemental tables 9,10). For birthing parents, from 6 weeks postdelivery, there were fewer AEs in the azithromycin arm than the placebo arm (1.4% compared with 2.3%). In comparison to birthing parents, where the frequency of AEs reduced after 6 weeks (online supplemental table 9), the frequency of infant AEs increased slightly from 6 weeks to 12 months of age; however, it remained evenly distributed between study arms (azithromycin: 1.6%; placebo: 2.1%).

## Discussion

We found that intrapartum azithromycin reduced the incidence of infections in birthing parents from 1 week postdelivery to 6 months postdelivery, with the effect size decreasing at each subsequent time point. The greatest effect was observed at 1 week postdelivery, with a 69% reduction in infections overall and 75% reduction in SSTIs, representing absolute reductions of 1.8% for infections overall and 1.3% for SSTIs, corresponding to NNTs of 56 and 79, respectively. We observed a reduction in overall infant infections in the azithromycin group at 3 months of age (RR 0.79; 95% CI 0.63 to 0.99), equating to 27 NNT to prevent one infant infection. While the point estimate of infant SSTIs in the azithromycin group was reduced at all time points, 95% CIs included the possibility of no effect.

The impact of intrapartum azithromycin on infections in birthing parents observed in our study is consistent with findings from a 2024 systematic review and meta-analysis showing that intrapartum azithromycin prevented maternal sepsis, endometritis, surgical site infections and antibiotic use.^18^ However, individual large RCTs included in this systematic review had differing results. The PregnAnZI-2 study from The Gambia and Burkina Faso had a primary outcome of neonatal sepsis or death. It found that mastitis and puerperal fever were less common in the azithromycin group than the placebo group, but found no difference in maternal death, puerperal sepsis, malaria or antibiotic use.^17^ In comparison, the A-PLUS study, conducted in seven LMICs from Africa, Asia and Latin America, found strong evidence of a reduction in the incidence of maternal sepsis and death in the azithromycin group, which may be explained by being powered to detect maternal sepsis and death as a primary outcome and its subsequent larger sample size (29 278 vs 11 983).^16 17 39^

Evidence for the benefits of oral azithromycin during delivery on infant infections is mixed. The 2024 systematic review and meta-analysis looked at a variety of neonatal outcomes, showing evidence of its effect on skin infections, omphalitis and neonatal antibiotic use, but not for all-cause mortality, sepsis, conjunctivitis, neonatal malaria, otitis, readmission or prolongation of admission, intensive care unit admission and Apgar score at 1 min.^18^ The PregnAnZI-2 and A-PLUS studies did not show evidence for the intervention’s effect on neonatal sepsis and death, although the PregnAnZI-2 study showed benefit for preventing skin infections (OR 0.48; 95% CI 0.34 to 0.67) and infections overall (OR 0.68; 95% CI 0.56 to 0.83).^16 17^ Three observational studies, two in Finland and one in Israel, have explored the long-term association between intrapartum antibiotics and childhood infectious diseases with conflicting results.^7 40 41^ Differing results between studies may be explained by variation in infections included as outcomes and how these were diagnosed and/or defined, as well as epidemiological contexts. The proportion of caesarean sections also varied considerably between studies (ranging from 1.2% to 14.1%),^16 17 42^ with subgroup analyses from the A-PLUS study showing a larger benefit of intrapartum azithromycin for preventing maternal death or sepsis in vaginal deliveries but no difference by type of delivery for the outcome of stillbirth or neonatal death or sepsis within 4 weeks.^16^ Caesarean section was relatively common in our study (23.3%) with an associated high rate of non-study antibiotic use during admission, consistent with use observed in other studies (35.7% vs 40.8%).^43^

Our study is the first to observe a prolonged effect of azithromycin for preventing SSTIs and other infections in birthing parents as we were able to follow up participants for longer than previous intrapartum azithromycin studies (day 28/42 vs 1 year).^16 17^ The explanation for this observed prolonged effect of single-dose intrapartum azithromycin for preventing infant infections at 3 months, and infections in birthing parents up to 6 months, is unclear. This effect may be explained by reduced horizontal transmission of potentially pathogenic bacteria given the proven efficacy of single-dose intrapartum azithromycin on carriage of GBS, *S. aureus* and *S. pneumoniae* in the breast milk and nasopharynx of birthing parents and infants at 28 days,^14^ although evidence suggests these effects waned by 12 months of age.^44^ Bacterial infection is often preceded by carriage, and progression to clinical infection may be facilitated through the acquisition of new virulence determinants or mutations, as suggested by a 2024 Gambian analysis.^45^ Therefore, decreased carriage of these potential pathogens in both infants and birthing parents may have contributed to decreased rates of infection observed over long-term follow-up in this study. Another potential mechanism for the prolonged effect of protection against infection for infants at 3 months of age could be ongoing infant exposure in breast milk, with evidence showing that there are high concentrations of azithromycin secreted in breast milk for at least 4 weeks.^12^ Childhood infection and nutrition have a bidirectional relationship,^46^ and intrapartum azithromycin has been shown to have some benefits on infant growth, with a lower proportion of children aged 11–13 months exposed to azithromycin diagnosed with malnutrition based on mid upper arm circumference, compared with those exposed to placebo.^47^ However, our study did not observe large differences between groups for proportion of those wasted or underweight at any timepoint, and therefore, malnutrition is unlikely to explain the reduction in infant infections observed in the azithromycin arm at 3 months. Future studies and research could look at extended follow-up periods to confirm this prolonged effect, as well as possible mechanisms for this.

The benefits of intrapartum azithromycin need to be weighed against potential harms including AMR, microbiome dysbiosis, impact on the development of the infant immune system and drug side effects. As previously reported, intrapartum azithromycin was well tolerated in this study.^30^ Post hoc analyses of the PregnAnZI and PregnAnZI-2 studies of the nasopharyngeal and gut microbiome have shown initial impacts on microbiome diversity that resolved between 28 days and 4 months of age, and changes to composition at 12 months of age that converged by 3 years of age.^48 49^ Similarly, a study in Finland of vaginally delivered infants exposed to intrapartum antibiotics (not specified to be azithromycin) found ongoing differences in composition at 1 year, particularly reduced relative abundance of *E. coli* in the control group compared with azithromycin arm.^50^ The clinical implications of any impact on the microbiome are not clear.

Current evidence from Africa suggests that mass drug administration of azithromycin and intrapartum azithromycin is associated with increased resistance to azithromycin among a range of bacteria immediately after treatment.^51^ Additionally, findings from individual studies suggest that this effect may be prolonged, with increased macrolide resistance determinants in the gut resistome of children in treatment arms 60 months following treatment,^52^ as well as increased determinants of non-macrolide resistance 48 months after treatment.^53^ Conversely, follow-up of the PregnAnZI study in The Gambia observed a higher prevalence of azithromycin-resistant *S. aureus* 4 weeks after intrapartum azithromycin that returned to baseline 12 months after delivery.^44^ However, whether using azithromycin in labour generates the same or greater antibiotic selection pressure than community level intermittent mass drug administration remains to be determined. It is also unclear whether genotypic resistance patterns from carriage isolates relate to clinical AMR patterns, and two ancillary studies to the A-PLUS trial observed no difference in the frequency of multiple azithromycin-resistant organisms detected in parental or newborn infection culture specimens.^54 55^ Despite this, uncertainty around the impact of these interventions on the microbiome and resistome highlights the need for coordinated surveillance and research.

Application of our findings to clinical practice is supported by evaluation of the NNT. While the NNT is likely too high to support prophylactic treatment in the general population for maternal sepsis (NNT 420),^18^ the NNT for surgical site infections is more convincing (NNT 25–79)^18^ and The American College of Obstetricians and Gynaecologists’ guidelines now recommend consideration of prophylactic adjunct azithromycin for nonelective caesarean sections.^56^ However, the NNT may be reduced by targeted use based on risk factors or population characteristics, thereby minimising potential harms outlined earlier. Evidence of benefit for specific groups is currently limited^16 57^ and is an important area of future research that could inform development of decision support tools.

There are several limitations to our study in addition to those already discussed. Our findings may not be generalisable, as our study population had clinical and demographic features that are unlikely to be reflective of the general population of Fiji, including lower prevalence of diabetes and chorioamnionitis,^58 59^ lower rates of prematurity and low birth weight,^60^ and residence in urban and periurban areas; likely due to our eligibility criteria. Variation between studies with differing contexts and population characteristics suggests that these factors are likely to impact the efficacy of intrapartum azithromycin on infection outcomes, which is an important area for future research to better target and maximise the benefits of this intervention. Furthermore, we were dependent on self-report for some outcomes including antibiotic prescription, due to inconsistent availability of documentation by community health providers. Last, this study was not powered to detect these composite secondary outcomes nor the individual infection outcomes making these up. Given that we were not powered for these outcomes and the small numbers, caution is required in interpretation of the results. These results need to be corroborated in larger studies, noting that these findings were consistent with meta-analyses and similar studies described previously, although these studies had a shorter follow-up period.^16^,^18^

COVID-19 pandemic-related restrictions significantly impacted our study, including effects on recruitment and follow-up. Face-to-face visits and assessments of SSTIs for both infants and birthing parents were unable to occur and were replaced by phone ‘visits’; assessment of SSTIs by self-report and confirmation by examination of photos. Primary outcome sensitivity analyses showed that infant SSTI diagnosis was lower when the mode of assessment was photo or self-reported compared with physical examination, suggesting that these modes of assessment were less sensitive than physical examination. However, this underestimate in incidence would have been non-differential between study arms. COVID-19 restrictions caused disruption to health services, although hospitals remained open, and impacted medical record keeping used for primary and secondary outcome ascertainment. These multiple impacts of COVID-19 restrictions on outcome ascertainment may have led to underestimation of infection outcomes in both study arms, potentially resulting in underestimation of the absolute risk difference between groups, and subsequent NNT. However, the relative treatment effect should have remained the same despite these limitations.

Our trial demonstrated that intrapartum azithromycin prevented postpartum infections among birthing parents, including SSTIs and infant infections at specific time points, consistent with previous studies. Our trial has extended prior evidence by demonstrating these effects beyond the neonatal and postpartum period. The relatively low NNT to prevent SSTIs in birthing parents and infant infections overall observed in this study and others^18^ suggests that this intervention warrants further consideration. Further research to better characterise the overall impact of this intervention is required, especially clinical and longer-term public health impacts related to AMR, the microbiome and resistome. Additionally, research is needed to inform implementation of this approach such as identifying target populations, developing an ethical framework for use, economic evaluation and potential complex interventions incorporating water, sanitation and hygiene and infection control and prevention. Potential opportunity costs such as decreased investment in comprehensive long-term strengthening of the maternal and newborn care continuum must also be considered.

## Supplementary material

10.1136/bmjgh-2025-019851 online supplemental file 1

10.1136/bmjgh-2025-019851 online supplemental file 2

## Data Availability

Data are available on reasonable request.
