# Supplementary material for: The efficacy of a single dose of oral azithromycin in labour to prevent infections in infants and birthing parents in Fiji: secondary outcomes from a randomised controlled trial
Source: BMJ Glob Health. 2026 Mar 4;11(3):e019851. doi: 10.1136/bmjgh-2025-019851 (PMC12970096; doi:10.1136/bmjgh-2025-019851)
Supplement: online supplemental file 1 [file bmjgh-11-3-s001.docx]

### BMJ Global Health Author Reflexivity Statement

Adapted from Morton, B., Vercueil, A., Masekela, R., Heinz, E., Reimer, L., Saleh, S., Kalinga, C., Seekles, M., Biccard, B., Chakaya, J., Abimbola, S., Obasi, A. and Oriyo, N. (2022), Consensus statement on measures to promote equitable authorship in the publication of research from international partnerships. Anaesthesia, 77: 264-276. <https://doi.org/10.1111/anae.15597>

| **Study conceptualisation** | |
| --- | --- |
| 1. How does this study address local research and policy priorities? | Given the high rates of skin and other infant and maternal infections in Fiji, azithromycin during labour offered potential for improving maternal and infant outcomes in this context. Results from a 2015 Gambian study showed reductions in infant infections overall, and the particularly large reductions observed in skin infections suggested that this intervention may have an additional benefit in the Fijian context because of its high burden of SSTIs. |
| 1. How were local researchers involved in study design? | The Directors of Obstetrics and Paediatrics (at the time of study initiation) at Colonial War Memorial Hospital, Suva, Fiji and policy-makers at Fiji’s Ministry of Health and Medical Services were Investigators of the study. These researchers supported study conception and design through a day-long study design workshop in Suva, Fiji, as well as establishment of the study in-country, including relevant government and ethical approval processes. |
| **Research management** | |
| 1. How has funding been used to support the local research team(s)? | There is a local Fijian research team. This funding was used to employ the study staff. |
| **Data acquisition and analysis** | |
| 1. How are research staff who conducted data collection acknowledged? | - All members of the Fijian research team are mentioned in the acknowledgements section of the paper “We sincerely thank the participating families on the Bulabula MaPei study **and acknowledge the dedicated Fijian study staff for their outstanding achievement on completing study visits, despite considerable uncertainty and major disruptions during the COVID-19 pandemic – *vinaka vaka levu*.”** - Some members of the Fijian research team including SC, the local study doctor, and TR, the local project co-ordinator are study authors. |
| 1. How have members of the research partnership been provided with access to study data? | Yes, and further nested studies as part of this larger Bulabula MaPei study; either undertaken primarily and/or with significant contributions from, Fijian researchers are forthcoming. |
| 1. How were data used to develop analytical skills within the partnership? | Through SC’s employment as study doctor for the trial, she was funded to undertake further postgraduate studies in epidemiology and public health, and to conduct independent nested studies as part of this larger Bulabula MaPei study; with forthcoming papers and associated analyses. |
| **Data interpretation** | |
| 1. How have research partners collaborated in interpreting study data? | All Fijian collaborators were provided opportunity to contribute to manuscript drafts, and provided context specific advice and interpretation as required. |
| **Drafting and revising for intellectual content** | |
| 1. How were research partners supported to develop writing skills? | - Research partners presented results from the Bulabula MaPei study (including nested studies) in local and international forums including developing associated presentations and posters. - There are also forthcoming papers authored by Fijian partners from this study. |
| 1. How will research products be shared to address local needs? | - Policymakers in Fiji were included from the conception and design stage through to implementation of the study, and have been included throughout the analysis and publication process of papers associated with the study. - The methodology and results of the study have been shared at forums with Fijian government and health sector representatives, as well as at local research conferences. |
| **Authorship** | |
| 1. How is the leadership, contribution and ownership of this work by LMIC researchers recognised within the authorship? | 7 of 15 of the authors are Fijian, including the 2^nd^ and 3^rd^ author, and 2^nd^ senior author. |
| 1. How have early career researchers across the partnership been included within the authorship team? | Both MHN (1^st^ author) from MCRI Australia and SC (2^nd^ author) from the Ministry of Health and Medical Services Fiji are early career researchers. |
| 1. How has gender balance been addressed within the authorship? | 11 out of 15 authors are women, including the first eight authors, and two senior authors (IT and FMR). |
| **Training** | |
| 1. How has the project contributed to training of LMIC researchers? | The Bulabula MaPei study supported continued development of experienced Fijian research professionals such as the local study manager TR; providing additional expertise in conducting interventional studies in pregnancy. TR ensured that study staff received Good Clinical Practice training. It also supported training of the Fijian study doctor SC, and senior researchers involved with the study supported SC to pursue independent studies and research during the course of the study. |
| **Infrastructure** | |
| 1. How has the project contributed to improvements in local infrastructure? | Part of the project included providing funding for local capacity building for antibiotic sensitivity testing and qPCR on surveillance samples at Colonial War Memorial Hospital, Fiji. |
| **Governance** | |
| 1. What safeguarding procedures were used to protect local study participants and researchers? | - Senior Fijian clinicians and policymakers were investigators of this trial, and therefore had significant influence and responsibility for the study, for example providing steer around how study processes should adapt during periods of COVID-19 pandemic-related restrictions in Fiji. - The local study manager was Fijian, and there was frequent communication between MCRI Australia and the MCRI study site in Fiji for escalation of concerns. - A Data Safety Monitoring Board (DSMB) with Fijian representation was established, with a detailed Safety Plan in the case of adverse events, including quarterly unblinded reporting to the DSMB, and biannual meetings. |
