# Supplementary material for: The efficacy of a single dose of oral azithromycin in labour to prevent infections in infants and birthing parents in Fiji: secondary outcomes from a randomised controlled trial
Source: BMJ Glob Health. 2026 Mar 4;11(3):e019851. doi: 10.1136/bmjgh-2025-019851 (PMC12970096; doi:10.1136/bmjgh-2025-019851)
Supplement: online supplemental file 2 [file bmjgh-11-3-s002.docx]

Hume-Nixon et al. *The safety and efficacy of a single dose of oral azithromycin in labour to prevent infections in infants and birthing parents in Fiji: secondary outcomes from a randomised controlled trial.*

**SUPPLEMENTARY APPENDIX**

**Supplementary Textbox 1: Infant infections - Definitions**

| - *Overview* - Infant infections were defined as the occurrence of one or more of the following events: meningitis (all cause, lab confirmed and clinically suspected and treated); sepsis; lower respiratory tract infection; SSTI (as per primary outcome definition), diarrhoea; urinary tract infection; ophthalmia neonatorum; or fever, up to and including 12 months of age. - The definition for SSTIs of impetigo, furuncle, abscess, and cellulitis were the same for infants and birthing parents but infant SSTIs also included staphylococcal scalded skin syndrome and omphalitis. Diarrhoea, fever, and pneumonia were captured in the study in multiple ways, and the number of cases of diarrhoea, fever, and pneumonia were a total of all stated reporting mechanisms. - *Diarrhoea:* Captured in three different ways:  1. *Hospitalisation for diarrhoea*: a discharge diagnosis of acute diarrhoea or acute gastroenteritis by treating medical staff during a hospitalisation for diarrhoea, and/or as categorised as ‘acute diarrhoea’ (as opposed to ‘chronic diarrhoea’ of ≥14 days duration) based on admission notes by the study doctor. 2. *Reported outpatient treatment for diarrhoea*: participating mothers will be asked if their infants have been treated for diarrhoea as an outpatient, without hospitalisation. 3. *Diarrhoea as a symptom reported during study visit*: at each study visit, study staff will ask about ‘diarrhoea’ as a solicited non serious adverse event. The definition of diarrhoea is as per IMCI guidance[1], differing based on the age of the infant.  - *In visits 1-3*, when the infant is aged less than 2 months, this will be defined as: ‘If the stools have changed from the usual pattern and are many and watery (more water than faecal matter)’. The normally frequent or semi-solid stools of a breastfed baby are not diarrhoea. - *In visits 4-6*, when the infant is aged more than 2 months, this will be defined as ‘three or more loose or watery stools in a 24-hour-period’. - *Fever:* Captured in three different ways:  1. *Self-report* (at each study visit: any maternal report of fever in the infant since the previous study visit) 2. *Objective fever during a study visit* (defined as documented axillary temperature ≥37.5°C, measured using a digital thermometer by staff) 3. *Documented fever during hospital admission* (defined as a documented temperature ≥37.5°C in hospital admission records)  - *Lower Respiratory Tract Infection (LRTI):* Captured in two different ways:  1. *Reported outpatient treatment for pneumonia*: participating mothers will be asked if their infants have been treated for pneumonia as an outpatient, without hospitalisation. 2. *Hospitalisation for LRTI (pneumonia or bronchiolitis):*  - *pneumonia***:** Admissions records reviewed, and then classified according to the the WHO pneumonia definition[2]: If presence of both signs of fast breathing (respiratory rate equal or greater than 50 breaths/min in a child aged 2-11 months) and chest indrawing present. For those younger than 2 months, diagnosis of pneumonia or congenital pneumonia by medical staff. - Bronchiolitis as diagnosed by the treating doctor - *Meningitis*: based on WHO criteria definitions[3]; a suspected meningitis case with CSF examination showing abnormality associated with bacterial meningitis including  1. A turbid macroscopic appearance and/or; 2. Increased opening pressure (>180mm water), if available, and/or; 3. Pleocytosis (usually of polymorphonuclear (PMN) leukocytes); WBC counts >10 cells/mm3 and/or; 4. Increased protein concentration (>45mg/dl); and/or 5. Decreased glucose concentration (<45 mg/dl).  - *Sepsis*: According to International Pediatric Sepsis Consensus Conference Definition (Goldstein et al 2005) ‘SIRS in the presence of or as a result of suspected or proven infection’, based on the criteria for SIRS described in this definition, based on temperature, tachycardia, respiratory rate, and leukocyte count as per age-specific vital signs and laboratory variables[4]. - *Urinary Tract Infections*: Based on the WHO Hospital care for Children[2], based on microscopy of a ‘clean’ urine sample (either ‘clean-catch’ urine specimen, in-out urinary catheter, or supra-pubic bladder aspiration) with more than five white cells per high-power field, or a dipstick shows a positive result for leukocytes, or culture positive. - *Opthalmia neonatorum*: As diagnosed by the treating doctor. |
| --- |

**Supplementary Textbox 2: Infections in birthing parents - Definitions**

| - *Overview:* - Infections in birthing parents was defined as the occurrence of one or more of the following: mastitis; post-operative wound infection; maternal sepsis, puerperal sepsis; SSTIs (including impetigo, furuncle, abscess, cellulitis) or any diagnosis of meningitis, pneumonia, fever, abdominal or pelvic abscess, endometritis, urinary tract infection, pyelonephritis, or chorioamnionitis up to and including six weeks post-delivery. - Total SSTIs in birthing parents included impetigo, furuncle, abscess, cellulitis, mastitis, and post-operative wound infection. - Diagnoses of mastitis, post-operative wound infection, pneumonia, urinary tract infection, pyelonephritis, and fever were captured in multiple ways; through documented hospitalisations, clinical examination by study staff, and reported outpatient treatment, whereas other infections in birthing parents were based on diagnosis during hospital admission by treating medical staff. - *Pneumonia, urinary tract infection, and pyelonephritis:* Captured in two ways:  1. Reported outpatient treatment. 2. Hospitalisation for condition.  - *Mastitis and post-operative wound infection:* Captured in three ways:  1. Through clinical examination and diagnosis by study staff at scheduled study visits. 2. Hospitalisation for condition. 3. Self-reported outpatient treatment for these conditions.  - *Mastitis* is defined as: part of the breast (usually unilateral) becomes red, painful, swollen and hard, that may be accompanied by general symptoms of fever and malaise[5]. - *Post-operative wound infection* is defined as: presence of either superficial or deep incisional surgical-site (for caesarean section) infection characterised by cellulitis or erythema and induration around the incision or purulent discharge from the incision site with or without fever and includes necrotising fasciitis. - *Fever:* Captured in three different ways:  1. Self-report (at each study visit: any self-reported fever, since the previous study visit) 2. Objective fever during a study visit (defined as documented axillary temperature ≥38°C, measured using a digital thermometer by staff) 3. Documented fever during hospital admission (defined as a documented temperature ≥38°C in hospital admission records).  - *Abdominal or pelvic abscess, endometritis, and puerperal sepsis:* Based on a clinical discharge diagnosis during hospital admission by treating medical staff. - *Meningitis:* All-cause meningitis cases will be a hospital admission with a clinical diagnosis of meningitis. This will then be further divided into categories based on lab investigations, as per definitions of infant meningitis. - *Maternal sepsis* - *All-cause maternal sepsis* cases are a hospital admission of the mother with a clinical diagnosis of sepsis, excluding diagnoses of puerperal sepsis. This includes microbiologically confirmed sepsis. - *Microbiologically confirmed sepsis* will be defined as a hospital admission for suspected sepsis case that is microbiologically confirmed by growing (i.e. culturing) the pathogen by blood culture or from a normally sterile site (excluding cases of puerperal sepsis). |
| --- |

**Supplementary Textbox 3: Secondary outcome measures and ascertainment for harms**

Secondary safety and tolerability outcomes were also documented at each study visit through asking about symptoms, and hospitalisation. Additionally electronic hospital records of participants were searched to confirm hospitalisation and to identify other serious adverse events (SAEs), including a final search at the end of participant follow-up. An AE was any untoward, undesired or unexpected clinical event that occurred in a participant exposed to the investigational product, irrespective of whether it was related. AEs that resulted in death; were life-threatening; required inpatient hospitalisation (including admission to baby unit) or prolonged existing hospitalisation; and/or resulted in persistent or significant disability or incapacity, were classified as SAEs. A set of pre-specified symptoms of drug reactions associated with azithromycin were asked about at early study visits.

**Supplementary Figure 1: Study procedures for birthing parents and infants up to and including twelve months postpartum**


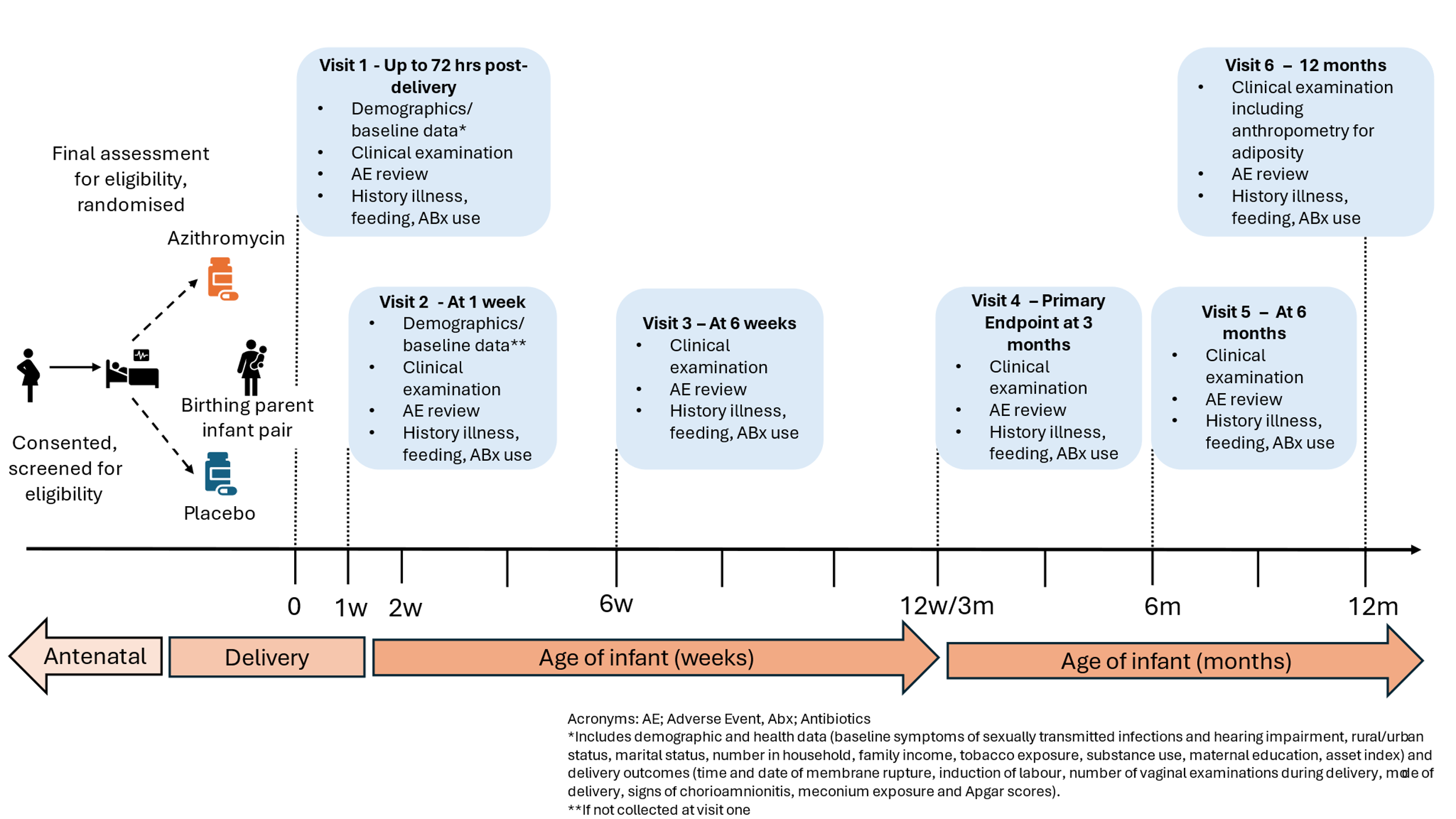


**Table S1: Number and percentage of birthing parents completing study visits and meeting criteria for complete cases analysis up to and including 12 months post-delivery.**

| **Visit** | **Time post-delivery** | **Study visit completion** | | **Complete case analysis** | |
| --- | --- | --- | --- | --- | --- |
|  |  | **Azithromycin** | **Placebo** | **Azithromycin** | **Placebo** |
| Visit one | Birth until discharge following delivery | 1053/1055 (99.8%) | 1055/1055 (100.0%) | 1046/1055 (99.1%) | 1053/1055 (99.8%) |
| Visit two | 1 week | 1018/1055 (96.5%) | 1023/1055 (97.0%) | 986/1055 (93.5%) | 995/1055 (94.3%) |
| Visit three | 6 weeks | 974/1055 (92.3%) | 975/1055 (92.4%) | 908/1055 (86.1%) | 917/1055 (86.9%) |
| Visit four | 3 months | 971/1055 (92.0%) | 975/1055 (92.4%) | 856/1055 (81.1%) | 875/1055 (82.9%) |
| Visit five | 6 months | 968/1055 (91.8%) | 954/1055 (90.4%) | 813/1055 (77.1%) | 818/1055 (77.5%) |
| Visit six | 12 months | 983/1055 (93.2%) | 989/1055 (93.7%) | 752/1055 (71.3%) | 779/1055 (73.8%) |

**Table S2: Number and percentage of infants completing study visits and meeting criteria for complete cases analysis up to and including 12 months post-delivery.**

| **Visit** | **Infant age** | **Study visit completion** | | **Complete case analysis** | |
| --- | --- | --- | --- | --- | --- |
|  |  | **Azithromycin*** | **Placebo** | **Azithromycin*** | **Placebo** |
| Visit one | Birth until discharge following delivery | 1058/1058 (100.0%) | 1063/1063 (100.0%) | 1035/1058 (97.8%) | 1043/1063 (98.1%) |
| Visit two | 1 week | 1021/1058 (96.5%) | 1028/1063 (96.7%) | 969/1058 (91.6%) | 987/1063 (92.9%) |
| Visit three | 6 weeks | 980/1058 (92.6%) | 983/1063 (92.5%) | 888/1058 (83.9%) | 907/1063 (85.3%) |
| Visit four | 3 months | 973/1058 (92.0%) | 988/1063 (92.9%) | 816/1058 (77.1%) | 855/1063 (80.4%) |
| Visit five | 6 months | 975/1058 (92.2%) | 963/1063 (90.6%) | 779/1058 (73.6%) | 797/1063 (75.0%) |
| Visit six | 12 months | 989/1058 (93.5%) | 998/1063 (93.9%) | 720/1058 (68.1%) | 750/1063 (70.6%) |

*One infant in azithromycin group withdrew before baseline data collected (e.g. date of birth, delivery outcome) so not included in study visit denominator

**Table S3: Characteristics of birthing parents, by treatment allocation**

|  | **Azithromycin (N=1055) n (%)*** | **Placebo (N=1055) n (%)*** |
| --- | --- | --- |
| **Sociodemographic** |  |  |
| Age in years, median (IQR) | 27.4 (23.1-31.9) | 27.4 (23.2-32.3) |
| Ethnicity |  |  |
| Other | 183 (17.3%) | 184 (17.4%) |
| iTaukei/Indigenous Fijian | 872 (82.7%) | 871 (82.6%) |
| Residential location |  |  |
| Rural | 67 (6.4%) | 67 (6.4%) |
| Urban | 452 (42.8%) | 436 (41.3%) |
| Peri-urban | 536 (50.8%) | 552 (52.3%) |
| Relationship status (N=1659) |  |  |
| Married or defacto | 728/831 (87.6%) | 741/828 (89.5%) |
| Sole parent^1^ | 103/831 (12.4%) | 87/828 (10.5%) |
| Level of education |  |  |
| Tertiary | 447 (42.4%) | 470 (44.5%) |
| Polytechnic/vocational | 4 (0.4%) | 1 (0.1%) |
| Completed secondary | 290 (27.5%) | 300 (28.4%) |
| Complete primary | 302 (28.6%) | 273 (25.9%) |
| Incomplete primary or none | 12 (1.1%) | 11 (1.0%) |
| Employment (N=2110) |  |  |
| Paid employment | 290 (27.5%) | 307 (29.1%) |
| Self-employed | 12 (1.1%) | 20 (1.9%) |
| Not in paid employment | 753 (71.4%) | 728 (69.0%) |
| Total number of household members, median (IQR) (N=2110) | 6.0 (4.0-8.0) | 6.0 (4.0-8.0) |
| Estimated weekly family income, $FJ median (IQR) (N=2107) | 300.0 (200.0-400.0) | 300.0 (200.0-400.0) |
| Relative to poverty line |  |  |
| At or above poverty line | 860/1052 (81.7%) | 846/1055 (80.2%) |
| Below poverty line | 192/1052 (18.3%) | 209/1055 (19.8%) |
| Cigarette use | 103 (9.8%) | 121 (11.5%) |
| Alcohol use | 55 (5.2%) | 62 (5.9%) |
| **Clinical characteristics** |  |  |
| Body mass index (BMI)^2^ (N=1292) |  |  |
| Mean (SD) | 30.7 (6.5) | 31.4 (7.3) |
| Normal weight (18.5-24.9) | 110/646 (17.0%) | 122/646 (18.9%) |
| Underweight (<18.5) | 12/646 (1.9%) | 8/646 (1.2%) |
| Pre-obesity (BMI 25.0-29.9) | 200/646 (31.0%) | 169/646 (26.2%) |
| Obesity class 1 & II (30.0-39.9) | 270/646 (41.8%) | 272/646 (42.1%) |
| Obese class III (BMI equal or greater than 40) | 54/646 (8.4%) | 75/646 (11.6%) |
| Diabetes mellitus |  |  |
| Gestational diabetes | 86/1050 (8.2%) | 85/1054 (8.1%) |
| Pre-existing diabetes | 1/1050 (0.1%) | 0/1054 (0.0%) |
| Pre-eclampsia | 11/1050 (1.0%) | 11/1054 (1.0%) |
| Hypertension | 21/1050 (2.0%) | 25/1054 (2.4%) |
| Cigarette use | 103 (9.8%) | 121 (11.5%) |
| Alcohol use | 55 (5.2%) | 62 (5.9%) |
| Total number of ANC visits, mean (SD) (N=1633) | 7.9 (2.8) | 8.0 (2.9) |
| Urinary tract infection in pregnancy | 30/811 (3.7%) | 33/818 (4.0%) |
| Anaemia^3^ | 259/812 (31.9%) | 246/817 (30.1%) |
| Treated syphilis in pregnancy^4^ | 36/812 (4.4%) | 48/819 (5.9%) |
| **Labour details** |  |  |
| Mode of delivery (N=2107) |  |  |
| Vaginal - non-instrumental | 807/1052 (76.7%) | 791/1055 (75.0%) |
| Vaginal - instrumental | 9/1052 (0.9%) | 10/1055 (0.9%) |
| Caesarean | 236/1052 (22.4%) | 254/1055 (24.1%) |
| Hours between rupture of membrane and delivery, median (IQR) | 1.5 (0.2-6.3) | 1.4 (0.2-6.9) |
| Induction of labour | 115/862 (13.3%) | 109/872 (12.5%) |
| Fever during delivery | 6/997 (0.6%) | 6/992 (0.6%) |
| Chorioamnionitis based on diagnostic criteria^5^ | 2/996 (0.2%) | 1/992 (0.1%) |
| Treated with other antibiotics during admission | 355/1049 (33.8%) | 396/1054 (37.6%) |
| Vomited after administration of Investigational Product | 10 (0.9%) | 5 (0.5%) |
| **Pregnancy outcomes** |  |  |
| Hours between treatment and delivery, median (IQR) (N=2107) | 10.2 (3.8-32.6) | 11.2 (3.9-34.3) |
| 72 hours or greater between administration of study drug and delivery | 125/1052 (11.9%) | 131/1055 (12.4%) |
| Multiple delivery (N=2107)** | 6/1052 (0.6%) | 8/1055 (0.8%) |

*Unless otherwise specified

**Two birthing parents had missing delivery data as they were withdrawn prior to this being collected

^1^Sole parent category includes single and widowed

^2^BMI categories as per WHO <18.5: Underweight; 18.5-24.9: Normal weight; 25.0-29.9: Pre-obesity; 30.0-39.9: Obesity class I & II; >40: Obesity class III.

BMI calculated based on maternal weight pre-delivery.

^3^ <11.0 d/L as per WHO. Ref: *Prevalence of anaemia in pregnant women (aged 15-49) (%): World Health Organization; 2021 [cited 2021 Aug 25]. Available from:* [*https://www.who.int/data/gho/indicator-metadata-registry/imr-details/4552*](https://www.who.int/data/gho/indicator-metadata-registry/imr-details/4552)

^4^Completed treatment defined as 3 doses of penicillin given if VDRL or TPHA positive. Please note that this is based on treatment criteria in Fiji.

^5^Chorioamnionitis based on fever (defined as a documented temperature ≥38°C in hospital admission records) plus any of the following 1) Fetal tachycardia >160bpm 2) maternal white blood cell count >15.3) Purulent vaginal discharge (adapted from ACOG 2017’s committee recommendations. Ref: *Committee Opinion No. 712: Intrapartum Management of Intraamniotic Infection. Obstet Gynecol. 2017;130(2), e95–101.*

**Table S4: Infant characteristics by treatment allocation**

|  | **Azithromycin**  **(N=1059)** n (%)* | **Placebo**  **(N=1063)** n (%)* |
| --- | --- | --- |
| Sex (N=2121) |  |  |
| Female | 531/1058 (50.2%) | 504/1063 (47.4%) |
| Apgar score at birth (N=2117) |  |  |
| 1-6 | 49/1056 (4.6%) | 35/1061 (3.3%) |
| 7-10 | 1007/1056 (95.4%) | 1026/1061 (96.7%) |
| Birthweight (N=2106) |  |  |
| Mean, grams (SD) | 3441.0 (493.9) | 3422.6 (496.8) |
| Low birth weight <2500g | 26/1050 (2.5%) | 23/1056 (2.2%) |
| Gestational age (N=2109) |  |  |
| Mean, weeks (SD) | 39.7 (1.4) | 39.5 (1.6) |
| Preterm birth (<37w) | 29/1051 (2.8%) | 35/1058 (3.3%) |
| Stillbirth (N=2121) | 1/1058 (0.1%) | 1/1063 (0.1%) |
| Admitted to neonatal unit following delivery (N=2121) | 37/1058 (3.5%) | 27/1063 (2.5%) |
| Clinically suspected chorioamnionitis in birthing parent (N=2121) | 13/1058 (1.2%) | 16/1063 (1.5%) |
| Meconium exposure (N= 1661) | 143/827 (17.3%) | 134/834 (16.1%) |
| Neonatal death (within 28 days) (N=2121) | 2/1058 (0.2%) | 3/1063 (0.3%) |
| **Exclusive breastfeeding** |  |  |
| At discharge following delivery | 1043/1053 (99.1%) | 1056/1062 (99.4%) |
| At 1 week | 914/1011 (90.4%) | 884/1019 (86.8%) |
| At 6 weeks | 649/964 (67.3%) | 652/970 (67.2%) |
| At 3 months | 535/952 (56.2%) | 540/972 (55.6%) |
| At 6 months | 240/951 (25.2%) | 233/946 (24.6%) |
| **Underweight**** |  |  |
| At discharge following delivery | 25/986 (2.5%) | 25/996 (2.5%) |
| At 1 week | 28/708 (4.0%) | 26/735 (3.5%) |
| At 6 weeks | 16/465 (3.4%) | 17/480 (3.5%) |
| At 3 months | 10/378 (2.6%) | 8/428 (1.9%) |
| At 6 months | 11/401 (2.7%) | 10/412 (2.4%) |
| At 12 months | 11/385 (2.9%) | 11/387 (2.8%) |
| **Wasting**** |  |  |
| At discharge following delivery | 59/1013 (5.8%) | 65/1022 (6.4%) |
| At 1 week | 90/703 (12.8%) | 95/732 (13.0%) |
| At 6 weeks | 20/464 (4.3%) | 24/480 (5.0%) |
| At 3 months | 28/378 (7.4%) | 22/428 (5.1%) |
| At 6 months | 19/401 (4.7%) | 24/409 (5.9%) |
| At 12 months | 21/383 (5.5%) | 12/385 (3.1%) |
| **Received all vaccinations** |  |  |
| After birth*** | 1040/1053 (98.8%) | 1050/1062 (98.9%) |
| 6 week vaccinations | 963/968 (99.5%) | 966/972 (99.4%) |
| 10 week vaccinations | 951/952 (99.9%) | 964/972 (99.2%) |
| 14 week vaccinations | 941/952 (98.8%) | 941/948 (99.3%) |
| 12 month vaccinations | 933/942 (99.0%) | 945/957 (98.7%) |

* Unless otherwise specified. Noting that one infant in azithromycin group withdrew before baseline data collected, so for most variables 1058 was the denominator

** Using WHO child growth standards (based on age) *Wasted*: Weight-for-length z-scores <-2; *Underweight*: Weight-for-age z-scores <-2. Ref: *Interpreting Growth Indicators. Geneva, Switzerland: World Health Organization; 2008.*

*** BCG vaccine and Hepatitis B vaccine

**Table S5: Characteristics of birthing parents and infants by whether included in complete case analysis**

|  | **Visit 1 (discharge following delivery)** | | **Visit 2 (one week)** | | **Visit 3 (six weeks)** | | **Visit 4 (three months)** | | **Visit 5 (six months)** | | **Visit 6 (twelve months)** | |
| --- | --- | --- | --- | --- | --- | --- | --- | --- | --- | --- | --- | --- |
|  | **Not included in CCA, n (%)** | **Included in CCA, n (%)** | **Not included in CCA, n (%)** | **Included in CCA, n (%)** | **Not included in CCA, n (%)** | **Included in CCA, n (%)** | **Not included in CCA, n (%)** | **Included in CCA, n (%)** | **Not included in CCA, n (%)** | **Included in CCA, n (%)** | **Not included in CCA, n (%)** | **Included in CCA, n (%)** |
| **All birthing parents** | 11 (0.5%) | 2,099 (99.5%) | 129 (6.1%) | 1,981 (93.9%) | 285 (13.5%) | 1,825 (86.5%) | 379 (18.0%) | 1,731 (82.0%) | 479 (22.7%) | 1,631 (77.3%) | 579 (27.4%) | 1,531 (72.6%) |
| Age in years, median (IQR) | 28.8 | 27.4 | 25.1 | 27.5 | 25.7 | 27.6 | 26.2 | 27.7 | 26.2 | 27.8 | 26.2 | 27.9 |
| Ethnicity |  |  |  |  |  |  |  |  |  |  |  |  |
| Other | 2 (18.2%) | 365 (17.4%) | 19 (14.7%) | 348 (17.6%) | 42 (14.7%) | 325 (17.8%) | 53 (14.0%) | 314 (18.1%) | 68 (14.2%) | 299 (18.3%) | 80 (13.8%) | 287 (18.7%) |
| iTaukei/Indigenous Fijian | 9 (81.8%) | 1,734 (82.6%) | 110 (85.3%) | 1,633 (82.4%) | 243 (85.3%) | 1,500 (82.2%) | 326 (86.0%) | 1,417 (81.9%) | 411 (85.8%) | 1,332 (81.7%) | 499 (86.2%) | 1,244 (81.3%) |
| Residential location |  |  |  |  |  |  |  |  |  |  |  |  |
| Rural | 0 (0.0%) | 134 (6.4%) | 11 (8.5%) | 123 (6.2%) | 20 (7.0%) | 114 (6.2%) | 27 (7.1%) | 107 (6.2%) | 38 (7.9%) | 96 (5.9%) | 42 (7.3%) | 92 (6.0%) |
| Urban | 4 (36.4%) | 884 (42.1%) | 49 (38.0%) | 839 (42.4%) | 111 (38.9%) | 777 (42.6%) | 144 (38.0%) | 744 (43.0%) | 183 (38.2%) | 705 (43.2%) | 231 (39.9%) | 657 (42.9%) |
| Peri-urban | 7 (63.6%) | 1,081 (51.5%) | 69 (53.5%) | 1,019 (51.4%) | 154 (54.0%) | 934 (51.2%) | 208 (54.9%) | 880 (50.8%) | 258 (53.9%) | 830 (50.9%) | 306 (52.8%) | 782 (51.1%) |
| Estimated weekly family income, $FJ median (IQR) (N=2107) | 400.00 | 300.00 | 275.00 | 300.00 | 250.00 | 300.00 | 250.00 | 300.00 | 250.00 | 300.00 | 260.00 | 300.00 |
| Cigarette use | 1 (9.1%) | 223 (10.6%) | 18 (14.0%) | 206 (10.4%) | 37 (13.0%) | 187 (10.2%) | 46 (12.1%) | 178 (10.3%) | 58 (12.1%) | 166 (10.2%) | 72 (12.4%) | 152 (9.9%) |
| Mode of delivery (N=2107) |  |  |  |  |  |  |  |  |  |  |  |  |
| Vaginal - non-instrumental | 6 (75.0%) | 1,592 (75.8%) | 103 (81.7%) | 1,495 (75.5%) | 218 (77.3%) | 1,380 (75.6%) | 294 (78.2%) | 1,304 (75.3%) | 374 (78.6%) | 1,224 (75.0%) | 454 (78.8%) | 1,144 (74.7%) |
| Vaginal - instrumental | 0 (0.0%) | 19 (0.9%) | 2 (1.6%) | 17 (0.9%) | 3 (1.1%) | 16 (0.9%) | 3 (0.8%) | 16 (0.9%) | 3 (0.6%) | 16 (1.0%) | 3 (0.5%) | 16 (1.0%) |
| Caesarean section | 2 (25.0%) | 488 (23.2%) | 21 (16.7%) | 469 (23.7%) | 61 (21.6%) | 429 (23.5%) | 79 (21.0%) | 411 (23.7%) | 99 (20.8%) | 391 (24.0%) | 119 (20.7%) | 371 (24.2%) |
| Hours between rupture of membrane and delivery, median (IQR) | 1.5 | 1.5 | 2.1 | 1.5 | 1.3 | 1.6 | 1.4 | 1.6 | 1.6 | 1.5 | 1.6 | 1.5 |
| **All newborns** | 44 (2.1%) | 2,078 (97.9%) | 166 (7.8%) | 1,956 (92.2%) | 327 (15.4%) | 1,795 (84.6%) | 451 (21.3%) | 1,671 (78.7%) | 546 (25.7%) | 1,576 (74.3%) | 652 (30.7%) | 1,470 (69.3%) |
| Sex (N=2121) |  |  |  |  |  |  |  |  |  |  |  |  |
| Female | 14 (32.6%) | 1,021 (49.1%) | 63 (38.2%) | 972 (49.7%) | 138 (42.3%) | 897 (50.0%) | 191 (42.4%) | 844 (50.5%) | 241 (44.2%) | 794 (50.4%) | 286 (43.9%) | 749 (51.0%) |
| Apgar score at birth (N=2117) |  |  |  |  |  |  |  |  |  |  |  |  |
| 1-6 | 15 (37.5%) | 69 (3.3%) | 19 (11.7%) | 65 (3.3%) | 24 (7.4%) | 60 (3.3%) | 29 (6.5%) | 55 (3.3%) | 31 (5.7%) | 53 (3.4%) | 34 (5.2%) | 50 (3.4%) |
| 7-10 | 25 (62.5%) | 2,008 (96.7%) | 143 (88.3%) | 1,890 (96.7%) | 299 (92.6%) | 1,734 (96.7%) | 418 (93.5%) | 1,615 (96.7%) | 511 (94.3%) | 1,522 (96.6%) | 614 (94.8%) | 1,419 (96.6%) |
| Birthweight (N=2106). Mean, grams (SD) | 3096.7 (739.0) | 3437.9 (487.8) | 3363.9 (541.7) | 3437.3 (491.1) | 3437.8 (519.5) | 3430.7 (491.0) | 3450.9 (506.3) | 3426.7 (492.4) | 3452.5 (504.6) | 3424.7 (492.1) | 3438.6 (495.6) | 3428.8 (495.4) |
| Low birth weight <2500g | 7 (18.4%) | 42 (2.0%) | 7 (4.4%) | 42 (2.2%) | 11 (3.4%) | 38 (2.1%) | 11 (2.5%) | 38 (2.3%) | 12 (2.2%) | 37 (2.4%) | 14 (2.2%) | 35 (2.4%) |
| Gestational age (N=2109) Mean, weeks (SD) | 38.7 (2.1) | 39.6 (1.5) | 39.5 (1.7) | 39.6 (1.5) | 39.7 (1.6) | 39.6 (1.5) | 39.7 (1.6) | 39.6 (1.5) | 39.7 (1.6) | 39.6 (1.5) | 39.7 (1.6) | 39.6 (1.5) |
| Preterm birth (<37w) | 8 (21.1%) | 56 (2.7%) | 11 (6.9%) | 53 (2.7%) | 15 (4.7%) | 49 (2.7%) | 17 (3.8%) | 47 (2.8%) | 18 (3.3%) | 46 (2.9%) | 23 (3.6%) | 41 (2.8%) |
| Exclusive breastfeeding | 37 (97.4%) | 2,062 (99.3%) | 67 (90.5%) | 1,731 (88.5%) | 85 (61.2%) | 1,216 (67.7%) | 150 (59.3%) | 925 (55.4%) | 89 (27.7%) | 384 (24.4%) | 12 (2.8%) | 13 (0.9%) |
| Wasting* | 0 (.%) | 124 (6.1%) | 8 (33.3%) | 177 (12.5%) | 2 (5.4%) | 42 (4.6%) | 4 (6.1%) | 46 (6.2%) | 10 (8.8%) | 33 (4.7%) | 8 (5.0%) | 25 (4.1%) |

Acronyms, Abbreviations: CCA: Complete case analysis; IQR: Interquartile Range; SD: Standard Deviation; w:weeks

*Using WHO child growth standards (based on age) *Wasted*: Weight-for-length z-scores <-2; *Underweight*: Weight-for-age z-scores <-2. Ref: *Interpreting Growth Indicators. Geneva, Switzerland: World Health Organization; 2008.*

**Table S6: Cumulative incidence of all infant infections up to and including 12 months of age by study arm – all cases**

|  | **Visit 1 (discharge following delivery)** | | | | **Visit 2 (one week)** | | | | **Visit 3 (six weeks)** | | | | **Visit 4 (three months)** | | | | **Visit 5 (six months)** | | | | **Visit 6 (twelve months)** | | | |
| --- | --- | --- | --- | --- | --- | --- | --- | --- | --- | --- | --- | --- | --- | --- | --- | --- | --- | --- | --- | --- | --- | --- | --- | --- |
|  | **AZI n/1059 (%)** | **PLA n/1063 (%)** | **RR (95% CI)** | **p-value** | **AZI n/1059 (%)** | **PLA n/1063 (%)** | **RR (95% CI)** | **p-value** | **AZI n/1059 (%)** | **PLA n/1063 (%)** | **RR (95% CI)** | **p-value** | **AZI n/1059 (%)** | **PLA n/1063 (%)** | **RR (95% CI)** | **p-value** | **AZI n/1059 (%)** | **PLA n/1063 (%)** | **RR (95% CI)** | **p-value** | **V6: AZI n/1059 (%)** | **PLA n/1063 (%)** | **RR (95% CI)** | **p-value** |
| **Infant infections incl. SSTIs (total)** | 34 (3.21) | 34 (3.20) | 1.00 (0.63-1.60) | 0.99 | 50 (4.72) | 54 (5.08) | 0.93 (0.64-1.35) | 0.70 | 88 (8.31) | 94 (8.84) | 0.94 (0.71-1.24) | 0.66 | 157 (14.83) | 183 (17.22) | 0.86 (0.71-1.05) | 0.13 | 307 (28.99) | 336 (31.61) | 0.91 (0.80-1.04) | 0.18 | 536 (50.61) | 585 (55.03) | 0.92 (0.85-0.99)* | 0.034* |
| Meningitis (total) | 1 (0.09) | 4 (0.38) | NA | NA | 3 (0.28) | 4 (0.38) | NA | NA | 4 (0.38) | 5 (0.47) | NA | NA | 6 (0.57) | 7 (0.66) | NA | NA | 7 (0.66) | 7 (0.66) | NA | NA | 10 (0.94) | 7 (0.66) | NA | NA |
| Sepsis | 3 (0.28) | 1 (0.09) | NA | NA | 6 (0.57) | 4 (0.38) | NA | NA | 7 (0.66) | 5 (0.47) | NA | NA | 8 (0.76) | 5 (0.47) | NA | NA | 10 (0.94) | 6 (0.56) | NA | NA | 11 (1.04) | 7 (0.67) | NA | NA |
| Lower respiratory tract infection | 25 (2.36) | 22 (2.07) | NA | NA | 28 (2.64) | 23 (2.16) | NA | NA | 33 (3.116) | 29 (2.73) | NA | NA | 45 (4.25) | 39 (3.67) | NA | NA | 85 (8.03) | 79 (7.43) | NA | NA | 164 (15.49) | 151 (14.21) | NA | NA |
| Diarrhoea | 0 | 0 | NA | NA | 2 (0.19) | 4 (0.376) | NA | NA | 6 (0..57) | 7 (0.659) | NA | NA | 8 (0.76) | 12 (1 .13) | NA | NA | 20 (1.89) | 19 (1.79) | NA | NA | 34 (3.21) | 35 (3.29) | NA | NA |
| Urinary tract infection | 4 (0.38) | 5 (0.47) | NA | NA | 6 (0.57) | 6 (0.56) | NA | NA | 9 (0.85) | 9 (0.85) | NA | NA | 9 (0.85) | 9 (0.85) | NA | NA | 10 (0.94) | 9 (0.85) | NA | NA | 10 (0.94) | 9 (0.85) | NA | NA |
| Ophthalmia neonatorum | 0 | 0 | NA | NA | 0 | 0 | NA | NA | 0 | 1 (0.09) | NA | NA | 0 | 1 (0.09) | NA | NA | 0 | 1 (0.09) | NA | NA | 0 | 1 (0.09) | NA | NA |
| Fever | 9 (0.85) | 9 (0.85) | NA | NA | 18 (1.70) | 16 (1.51) | NA | NA | 37 (3.49) | 37 (3.48) | NA | NA | 69 (6.52) | 76 (7.15) | NA | NA | 170 (16.05) | 192 (18.06) | NA | NA | 395 (37.30) | 467 (43.93) | NA | NA |
| Infant SSTIs (total) | 1 (0.09) | 2 (0.19) | 0.50 (0.05-5.53) | 0.57 | 5 (0.47) | 12 (1.13) | 0.42 (0.15-1.19) | 0.10 | 21 (1.98) | 26 (2.45) | 0.81 (0.46-1.43) | 0.47 | 59 (5.57) | 77 (7.24) | 0.77 (0.55-1.07) | 0.12 | 127 (11.99) | 127 (13.73) | 0.87 (0.70-1.09) | 0.22 | 198 (18.70) | 207 (19.47) | 0.96 (0.80-1.14) | 0.61 |
| Omphalitis | 0 | 1 (0.094) | NA | NA | 2 (0.189) | 5 (0.47) | NA | NA | 5 (0.47) | 5 (0.47) | NA | NA | 5 (0.47) | 8 (0.75) | NA | NA | NA | NA | NA | NA | NA | NA | NA | NA |
| Impetigo | 0 | 0 | NA | NA | 0 | 2 (0.19) | NA | NA | 4 (0.38) | 5 (0.47) | NA | NA | 14 (1.32) | 17 (1.60) | NA | NA | 39 (3.68) | 40 (0.04) | NA | NA | 61 (5.76) | 62 (5.83) | NA | NA |
| Furuncle | 1 (0.094) | 1 (0.09) | NA | NA | 3 (0.283) | 5 (0.47) | NA | NA | 8 (0.76) | 13 (1.22) | NA | NA | 25 (0.02) | 37 (3.48) | NA | NA | 61 (5.76) | 71 (6.68) | NA | NA | 98 (9.25) | 101 (9.50) | NA | NA |
| Abscess | 0 | 0 | NA | NA | 0 | 1 (0.09) | NA | NA | 4 (0.38) | 4 (0.38) | NA | NA | 21 (1.98) | 23 (2.16) | NA | NA | 43 (4.06) | 49 (4.61) | NA | NA | 58 (5.48) | 67 (6.30) | NA | NA |
| Cellulitis | 0 | 0 | NA | NA | 0 | 0 | NA | NA | 0 | 0 | NA | NA | 2 (0.19) | 0 | NA | NA | 4 (0.378) | 1 (0.09) | NA | NA | 10 (0.94) | 3 (0.38) | NA | NA |
| SSSS | 0 | 0 | NA | NA | 0 | 0 | NA | NA | 0 | 0 | NA | NA | 0 | 0 | NA | NA | 0 | 0 | NA | NA | 0 | 0 | NA | NA |

Acronyms, Abbreviations: AZI: Azithromycin; PLA: Placebo; NA: Not applicable; SSTIs: Skin and soft tissue infections; SSSS: Staphylococcal scaled skin syndrome

*95% CI does not include null value

**Table S7: Cumulative incidence of infections in birthing parents up to and including 12 months post-delivery by study arm – all cases**

|  | **Visit 1 (discharge following delivery)** | | | | **Visit 2 (one week)** | | | | **Visit 3 (six weeks)** | | | | **Visit 4 (three months)** | | | | **Visit 5 (six months)** | | | | **Visit 6 (twelve months)** | | | |
| --- | --- | --- | --- | --- | --- | --- | --- | --- | --- | --- | --- | --- | --- | --- | --- | --- | --- | --- | --- | --- | --- | --- | --- | --- |
|  | **AZI n/1055) (%)** | **PLA n/1055) (%)** | **RR (95% CI)** | **p-value** | **AZI n/1055) (%)** | **PLA n/1055) (%)** | **RR (95% CI)** | **p-value** | **AZI n/1055) (%)** | **PLA n/1055) (%)** | **RR (95% CI)** | **p-value** | **AZI n/1055) (%)** | **PLA n/1055) (%)** | **RR (95% CI)** | **p-value** | **AZI n/1055) (%)** | **PLA n/1055) (%)** | **RR (95% CI)** | **p-value** | **AZI n/1055) (%)** | **PLA n/1055) (%)** | **RR (95% CI)** | **p-value** |
| **Infections in birthing parents incl. SSTIs (total)** | 3 (0.28) | 5 (0.47) | 0.60 (0.14-2.50) | 0.48 | 7 (0.66) | 25 (2.37) | 0.28 (0.12-0.64)* | 0.003* | 19 (1.80) | 47 (4.45) | 0.40 (0.24-0.68)* | 0.001* | 28 (2.65) | 52 (4.93) | 0.54 (0..34-0.85)* | 0.007* | 50 (4.74) | 80 (7.58) | 0.63 (0.44-0.88)* | 0.007* | 121 (11.47) | 164 (15.55) | 0.74 (0.59-0.92)* | 0.006* |
| Meningitis | 0 | 0 | NA | NA | 0 | 1 (0.09) | NA | NA | 0 | 1 (0.09) | NA | NA | 0 | 1 (0.09) | NA | NA | 0 | 1 (0.09) | NA | NA | 0 | 1 (0.09) | NA | NA |
| Sepsis | 0 (0.09) | 0 (0.09) | NA | NA | 1, (0.09) | 1 (0.09) | NA | NA | 1 (0.09) | 2, (0.19) | NA | NA | 1 (0.09) | 3 (0.28) | NA | NA | 1 (0.09) | 3 (0.28) | NA | NA | 1 (0.09) | 3 (0.28) | NA | NA |
| Puerperal sepsis | 0 | 0 | NA | NA | 0 | 0 | NA | NA | 0 | 0 | NA | NA | 0 | 0 | NA | NA | 0 | 0 | NA | NA | 0 | 0 | NA | NA |
| Pneumonia | 0 | 1 (0.09) | NA | NA | 0 | 1 (0.09) | NA | NA | 0 | 1 (0.09) | NA | NA | 2 (0.19) | 2 (0.19) | NA | NA | 4 (0.38) | 5 (0.47) | NA | NA | 6 (0.57) | 9 (0.85) | NA | NA |
| Abdominal or pelvic abscess | 0 | 0 | NA | NA | 0 | 0 | NA | NA | 0 | 0 | NA | NA | 0 | 0 | NA | NA | 0 | 0 | NA | NA | 0 | 0 | NA | NA |
| Endometritis | 0 | 0 | NA | NA | 0 | 0 | NA | NA | 0 | 0 | NA | NA | 0 | 0 | NA | NA | 0 | 0 | NA | NA | 0 | 0 | NA | NA |
| Urinary tract infection | 0 | 0 | NA | NA | 0 | 1 (0.09) | NA | NA | 0 | 1 (0.09) | NA | NA | 1 (0.09) | 1 (0.09) | NA | NA | 2 (0.19) | 1 (0.09) | NA | NA | 2 (0.19) | 2 (0.19) | NA | NA |
| Pyelonephritis | 0 | 0 | NA | NA | 0 | 0 | NA | NA | 0 | 0 | NA | NA | 0 | 0 | NA | NA | 0 | 0 | NA | NA | 0 | 0 | NA | NA |
| Chorioamnionitis | 1 (0.09) | 0 | NA | NA | 1 (0.09) | 0 | NA | NA | 1 (0.09) | 0 | NA | NA | 1 (0.09) | 0 | NA | NA | 1 (0.09) | 0 | NA | NA | 1 (0.09) | 0 | NA | NA |
| Fever | 2 (0.19) | 2 (0.19) | NA | NA | 3 (0.28) | 7 (0.66) | NA | NA | 9 (0.85) | 15 (1.42) | NA | NA | 13 (1.23) | 20 (1.90) | NA | NA | 32 (3.03) | 43 (4.08) | NA | NA | 98 (9.29) | 119 (11.28) | NA | NA |
| SSTIs in birthing parents (total) | 1 (0.09) | 3 (0.28) | 0.33 (0.03-3.19) | 0.34 | 4 (0.38) | 18 (0.66) | 0.22 (0.08-0.65)* | 0.006* | 10 (0.95) | 34 (3.22) | 0.29 (0.15-0.59)* | 0.001* | 17 (1.61) | 43 (4.08) | 0.40 (0.23-0.69)* | 0.001* | 24 (2.27) | 51 (4.83) | 0.47 (0.29-0.76)* | 0.002* | 30 (2.84) | 61 (5.78) | 0.49 (0.32-0.75)* | 0.001* |
| Mastitis | 0 | 0 | NA | NA | 0 (.) | 1 (0.09) | NA | NA | 0 (.) | 2 (0.19) | NA | NA | 0 | 2 (0.19) | NA | NA | 1 (0.09) | 2 (0.19) | NA | NA | 1 (0.09) | 2 (0.19) | NA | NA |
| Post-operative wound infection | 1 (0.09) | 0 | NA | NA | 3 (0.28) | 10 (0.95) | NA | NA | 3 (0.28) | 10 (1.23) | NA | NA | NA | NA | NA | NA | NA | NA | NA | NA | NA | NA | NA | NA |
| Impetigo | 0 | 1 (0.09) | NA | NA | 0 | 1 (0.09) | NA | NA | 0 (.) | 1 (0.095) | NA | NA | 0 | 1 (0.09) | NA | NA | 0 | 1 (0.28) | NA | NA | 0 | 3 (0.28) | NA | NA |
| Furuncle | 0 | 1 (0.09) | NA | NA | 0 | 3 (0.28) | NA | NA | 1 (0.09) | 14 (1.33) | NA | NA | 5 (0.47) | 20 (1.90) | NA | NA | 10 (0.95) | 25 (2.37) | NA | NA | 15 (1.42) | 34 (3.22) | NA | NA |
| Abscess | 0 | 1 (0.09) | NA | NA | 1 (0.09) | 3 (0.28) | NA | NA | 6 (0.57) | 6 (0.57) | NA | NA | 9 (0.85) | 9 (0.85) | NA | NA | 12 (1.14) | 9, (0.85) | NA | NA | 13 (1.23) | 11 (1.16) | NA | NA |
| Cellulitis | 0 | 0 | NA | NA | 0 | 0 | NA | NA | 0 | 0 | NA | NA | 0 | 0 | NA | NA | 0 | 1 (0.09) | NA | NA | 0 | 1 (0.09) | NA | NA |

Acronyms, Abbreviations: AZI: Azithromycin; PLA: Placebo; NA: Not applicable; SSTIs: Skin and soft tissue infections

*95% CI does not include null value

**Table S8: Cumulative incidence of antibiotic prescription in infant and birthing parents (throughout study duration) – all cases**

|  | **Visit 1 (discharge following delivery)** | | | | **Visit 2 (one week)** | | | | **Visit 3 (six weeks)** | | | | **Visit 4 (three months)** | | | | **Visit 5 (six months)** | | | | **Visit 6 (twelve months)** | | | |
| --- | --- | --- | --- | --- | --- | --- | --- | --- | --- | --- | --- | --- | --- | --- | --- | --- | --- | --- | --- | --- | --- | --- | --- | --- |
| **Infant participant prescribed antibiotics** | **AZI: n/1059 (%)** | **PLA: n/1063 (%)** | **RR (95% CI)** | **p-value** | **AZI: n/1059 (%)** | **PLA:n/1063 (%)** | **RR (95% CI)** | **p-value** | **AZI: n/1059 (%)** | **PLA:n/1063 (%)** | **RR (95% CI)** | **p-value** | **AZI: n/1059 (%)** | **PLA:n/1063 (%)** | **RR (95% CI)** | **p-value** | **AZI: n/1059 (%)** | **PLA:n/1063 (%)** | **RR (95% CI)** | **p-value** | **AZI: n/1059 (%)** | **PLA:n/1063 (%)** | **RR (95% CI)** | **p-value** |
|  | 170 (16.1) | 177 (16.7) | 0.96 (0.80-1.17) | 0.72 | 191 (18.0) | 214 (20.1) | 0.90 (0.75-1.07) | 0.23 | 207 (19.5) | 229 (21.5) | 0.91 (0.77-1.07) | 0.26 | 244 (23.0) | 275 (25.9) | 0.89 (0.77-1.04) | 0.13 | 358 (33.8) | 388 (36.5) | 0.92 (0.82-1.04) | 0.19 | 458 (43.2) | 506 (47.6) | 0.90 (0.82-0.99)* | 0.04* |
| **Birthing parent prescribed antibiotics** | **AZI n/1055 (%)** | **PLA n/1055 (%)** | **RR (95% CI)** | **p-value** | **AZI n/1055 (%)** | **PLA n/1055 (%)** | **RR (95% CI)** | **p-value** | **AZI n/1055 (%)** | **PLA n/1055 (%)** | **RR (95% CI)** | **p-value** | **AZI n/1055 (%)** | **PLA n/1055 (%)** | **RR (95% CI)** | **p-value** | **AZI n/1055 (%)** | **PLA n/1055 (%)** | **RR (95% CI)** | **p-value** | **AZI n/1055 (%)** | **PLA n/1055 (%)** | **RR (95% CI)** | **p-value** |
|  | 355 (33.6) | 396 (37.5) | 0.90 (0.80-1.01) | 0.06 | 366 (34.7) | 412 (39.1) | 0.89 (0.79-0.99)* | 0.04* | 373 (35.4) | 422 (40.0) | 0.88 (0.79-0.99)* | 0.03* | 381 (36.1) | 429 (40.7) | 0.89 (0.80-0.99)* | 0.03* | 392 (37.2) | 439 (41.6) | 0.89 (0.80-0.99)* | 0.04* | 404 (38.3) | 453 (42.9) | 0.89 (0.80-0.99)* | 0.03* |

Acronyms, Abbreviations: AZI: Azithromycin; PLA: Placebo

*95% CI does not include null value

**Table S9: Adverse events in birthing parents up to and including 12 months of age, by treatment allocation**

| **Adverse events** | **Azithromycin (N=1055)* n(%)** | **Placebo (N=1055)* n(%)** |
| --- | --- | --- |
| Total number of SAEs in birthing parents | 32 | 42 |
| Total number of birthing parents with SAEs | 32 (3.0%) | 39 (3.7%) |
| Fatal SAEs | 0 (0.0%) | 1 (0.1%) |
| Assessment of relationship to study drug |  |  |
| Unrelated | 30/32 (93.8%) | 36/42 (85.7%) |
| Unlikely | 2/32 (6.3%) | 5/42 (11.9%) |
| Possibly | 0/32 (0.0%) | 1/42 (2.4%) |
| Probably | 0/32 (0.0%) | 0/42 (0.0%) |
| Definitely | 0/32 (0.0%) | 0/42 (0.0%) |
| **Category of SAE up to and including 12 months post delivery** |  |  |
| Specific disorders of pregnancy | 10 (0.9%) | 14 (1.3%) |
| Infections (included in secondary outcome) | 3 (0.3%) | 10 (0.9%) |
| Other | 7 (0.7%) | 6 (0.6%) |
| SSTIs | 4 (0.4%) | 5 (0.5%) |
| Other infections (not included in secondary outcome) | 3 (0.3%) | 2 (0.2%) |
| Cardiovascular diseases | 1 (0.1%) | 3 (0.3%) |
| Blood system disorders | 2 (0.2%) | 0 (0.0%) |
| Hepatobiliary | 1 (0.1%) | 1 (0.1%) |
| Gastrointestinal | 1 (0.1%) | 0 (0.0%) |
| Respiratory | 0 (0.0%) | 1 (0.1%) |
| **Non-serious adverse events** |  |  |
| ***Up to 6 weeks post-delivery*** |  |  |
| Total number of AEs up to and including 6 weeks post-delivery | 100 | 82 |
| Total number of AEs up to and including 6 weeks post-delivery | 74 (7.0%) | 62 (5.9%) |
| **Category of non-serious AE up to and including 6 weeks** |  |  |
| Headache | 25 (2.4%) | 32 (3.0%) |
| Abdominal pain | 9 (0.9%) | 18 (1.7%) |
| Diarrhoea | 13 (1.2%) | 12 (1.1%) |
| Vomiting | 15 (1.4%) | 2 (0.2%) |
| Dizziness | 10 (0.9%) | 7 (0.7%) |
| Nausea | 7 (0.7%) | 1 (0.1%) |
| Fatigue | 7 (0.7%) | 2 (0.2%) |
| Pruritis/Itch | 4 (0.4%) | 2 (0.2%) |
| Arthralgia | 5 (0.5%) | 1 (0.1%) |
| Loss of appetite | 1 (0.1%) | 2 (0.2%) |
| Dyspepsia | 1 (0.1%) | 0 (0.0%) |
| Palpitations | 1 (0.1%) | 0 (0.0%) |
| Wheeze | 0 (0.0%) | 0 (0.0%) |
| Vaginitis | 1 (0.1%) | 0 (0.0%) |
| Visual disturbance | 1 (0.1%) | 1 (0.1%) |
| Taste disturbance | 0 (0.0%) | 1 (0.1%) |
| Numbness | 0 (0.0%) | 0 (0.0%) |
| Hearing impairment | 0 (0.0%) | 1 (0.1%) |
| ***From 6 weeks post-delivery to 12 months post-delivery*** |  |  |
| Total number of AEs after 6 weeks to 12 months post-delivery | 33 | 43 |
| Total number of AEs after 6 weeks to 12 months post-delivery | 15/1055 (1.4%) | 24/1055 (2.3%) |
| **Category of non-serious AEs after 6 weeks to 12 months post-delivery** |  |  |
| Abdominal pain | 6 (0.6%) | 14 (1.3%) |
| Diarrhoea | 6 (0.6%) | 11 (1.0%) |
| Nausea | 5 (0.5%) | 3 (0.3%) |
| Headache | 4 (0.4%) | 4 (0.4%) |
| Vomiting | 3 (0.3%) | 4 (0.4%) |
| Fatigue | 4 (0.4%) | 2 (0.2%) |
| Dizziness | 1 (0.1%) | 1 (0.1%) |
| Wheeze | 0 (0.0%) | 2 (0.2%) |
| Pruritis/Itch | 0 (0.0%) | 1 (0.1%) |
| Arthralgia | 0 (0.0%) | 0 (0.0%) |
| Loss of appetite | 1 (0.1%) | 0 (0.0%) |
| Dyspepsia | 1 (0.1%) | 0 (0.0%) |
| Palpitations | 1 (0.1%) | 0 (0.0%) |
| Vaginitis | 1 (0.1%) | 0 (0.0%) |
| Numbness | 0 (0.0%) | 1 (0.1%) |
| Visual disturbance | 0 (0.0%) | 0 (0.0%) |
| Taste disturbance | 0 (0.0%) | 0 (0.0%) |
| Hearing impairment | 0 (0.0%) | 0 (0.0%) |

* All birthing parents that received allocated dose

### **Table S10: Adverse events in infants up to and including 12 months, by treatment allocation**

| **Adverse events** | **Azithromycin (N=1059)* n(%)** | **Placebo (N=1063)* n(%)** |
| --- | --- | --- |
| Total number of infant SAEs | 290 | 297 |
| Total number of infant participants with SAEs | 268/1059 (25.3%) | 280/1063 (26.3%) |
| Fatal SAEs | 8 (0.8%) | 8 (0.8%) |
|  |  |  |
| Assessment of relationship to study drug |  |  |
| Unrelated | 278/290 (95.9%) | 291/297 (98.0%) |
| Unlikely | 12/290 (4.1%) | 6/297 (2.0%) |
| Possibly | 0/290 (0.0%) | 0/297 (0.0%) |
| Probably | 0/290 (0.0%) | 0/297 (0.0%) |
| Definitely | 0/290 (0.0%) | 0/297 (0.0%) |
|  |  |  |
| **SAE category** |  |  |
| Common neonatal conditions** | 155 (14.6%) | 172 (16.2%) |
| Infections (included in secondary outcome) | 83 (7.8%) | 64 (6.0%) |
| Infections (not included in secondary outcome) | 13 (1.2%) | 17 (1.6%) |
| SSTIs | 12 (1.1%) | 13 (1.2%) |
| Hepatobiliary condition | 10 (0.9%) | 11 (1.0%) |
| Death | 7 (0.7%) | 7 (0.7%) |
| Nervous system disorder | 4 (0.4%) | 2 (0.2%) |
| Blood system disorders | 2 (0.2%) | 1 (0.1%) |
| Gastrointestinal condition | 0 (0.0%) | 3 (0.3%) |
| Stillbirth | 1 (0.1%) | 1 (0.1%) |
| Cardiac condition | 0 (0.0%) | 2 (0.2%) |
| Metabolic and nutritional disorders | 1 (0.1%) | 1 (0.1%) |
| Respiratory | 1 (0.1%) | 1 (0.1%) |
| Other | 1 (0.1%) | 1 (0.1%) |
| Renal disorders | 0 (0.0%) | 1 (0.1%) |
| **Non-serious adverse events up to six weeks of age** |  |  |
| ***Up to 6 weeks of age*** |  |  |
| Total number of infant AEs | 3 | 9 |
| Total number of infant participants with AEs | 3 (0.3%) | 8 (0.8%) |
| **AE category** |  |  |
| Vomiting | 2 (0.2%) | 4 (0.4%) |
| Diarrhoea | 1 (0.1%) | 4 (0.4%) |
| Feeding difficulty | 0 (0.0%) | 1 (0.1%) |
| Urticaria | 0 (0.0%) | 0 (0.0%) |
| Angio-oedema | 0 (0.0%) | 0 (0.0%) |
| Eczema | 0 (0.0%) | 0 (0.0%) |
| Wheeze | 0 (0.0%) | 0 (0.0%) |
| Oral candidiasis | 0 (0.0%) | 0 (0.0%) |
| Hearing impairment | 0 (0.0%) | 0 (0.0%) |
| ***From 6 weeks to 12 months of age*** |  |  |
| Total number of AEs 6 weeks to 12 mths | 20 | 23 |
| Total number of participants with AEs 6 weeks to 12 mths | 17/1059 (1.6%) | 22/1063 (2.1%) |
| **AE category** |  |  |
| Diarrhoea | 15 (1.4%) | 15 (1.4%) |
| Wheeze | 2 (0.2%) | 4 (0.4%) |
| Vomiting | 2 (0.2%) | 3 (0.3%) |
| Feeding difficulty | 1 (0.1%) | 0 (0.0%) |
| Urticaria | 0 (0.0%) | 0 (0.0%) |
| Angio-oedema | 0 (0.0%) | 0 (0.0%) |
| Eczema | 0 (0.0%) | 0 (0.0%) |
| Oral candidiasis | 0 (0.0%) | 0 (0.0%) |
| Hearing impairment | 0 (0.0%) | 0 (0.0%) |

* All infants of birthing parents that received allocated dose (total number of births including stillbirths)

**Common neonatal conditions category includes: Physiological jaundice; meconium aspiration; Transient tachypnoea of the newborn (TTN); Birth asphyxia; Hypoxic ischaemic encephalopathy; Intraventricular haemorrhage; Feed intolerance; Prematurity; Hypoglycaemia; Hypothermia; Low birth weight; Bronchopulmonary Dysplasia; Necrotising Enterocolitis; Prolonged Rupture of Membranes (risk factor); Positive maternal syphilis serology with adequate treatment (risk factor); Respiratory distress syndrome; Suspected Maternal chorioamnionitis (risk factor); Positive Maternal Hepatitis B Serology (risk factor); Vitamin K deficiency bleeding; Cephalohematoma; Maternal Febrile Illness; Maternal Group B Streptococcus Carrier; Scalp Swelling-Caput Succedaneum; Shoulder Dystocia; Twin To Twin Syndrome; Umbilical Cord issues (no omphalitis); and Unknown Maternal Hepatitis B Serology.

**REFERENCES**

1. Integrated Management of Childhood Illness: distance learning course. Geneva, Switzerland: World Health Organization 2014.

2. Pocket book of hospital care for children: guidelines for the management of common childhood illnesses. 2^nd^ edition. World Health Organization 2013.

3. Laboratory Methods for the Diagnosis of Meningitis caused by *Neisseria meninigitidis*, *Streptococcus pneumoniae*, and *Haemophilus influenzae*. Geneva: World Health Organization 2011.

4. Goldstein B, Giroir B, Randolph A, et al. International pediatric sepsis consensus conference: definitions for sepsis and organ dysfunction in pediatrics. Pediatr Crit Care Med 2005;6:2-8.

5. Mastitis : causes and management. Geneva: World Health Organization 2000.
